# Methanol-based fixation is superior to buffered formalin for next-generation sequencing of DNA from clinical cancer samples

**DOI:** 10.1093/annonc/mdv613

**Published:** 2015-12-17

**Authors:** A. M. Piskorz, D. Ennis, G. Macintyre, T. E. Goranova, M. Eldridge, N. Segui-Gracia, M. Valganon, A. Hoyle, C. Orange, L. Moore, M. Jimenez-Linan, D. Millan, I. A. McNeish, J. D. Brenton

**Affiliations:** 1 Cancer Research UK Cambridge Institute, University of Cambridge, Cambridge; 2 Institute of Cancer Sciences, University of Glasgow, Glasgow; 3 Department of Pathology, Queen Elizabeth University Hospital, Glasgow; 4 Addenbrooke's Hospital, Cambridge University Hospital NHS Foundation Trust and National Institute for Health Research Cambridge Biomedical Research Centre, Cambridge; 5 Cancer Molecular Diagnostics Laboratory, Department of Oncology, University of Cambridge, Cambridge; 6 Department of Histopathology, Addenbrooke's Hospital, Cambridge, UK

**Keywords:** fixation, NBF, UMFIX, HGSOC, next-generation sequencing, copy-number abnormalities, SNVs

## Abstract

**Background:**

Next-generation sequencing (NGS) of tumour samples is a critical component of personalised cancer treatment, but it requires high-quality DNA samples. Routine neutral-buffered formalin (NBF) fixation has detrimental effects on nucleic acids, causing low yields, as well as fragmentation and DNA base changes, leading to significant artefacts.

**Patients and methods:**

We have carried out a detailed comparison of DNA quality from matched samples isolated from high-grade serous ovarian cancers from 16 patients fixed in methanol and NBF. These experiments use tumour fragments and mock biopsies to simulate routine practice, ensuring that results are applicable to standard clinical biopsies.

**Results:**

Using matched snap-frozen tissue as gold standard comparator, we show that methanol-based fixation has significant benefits over NBF, with greater DNA yield, longer fragment size and more accurate copy-number calling using shallow whole-genome sequencing (WGS). These data also provide a new approach to understand and quantify artefactual effects of fixation using non-negative matrix factorisation to analyse mutational spectra from targeted and WGS data.

**Conclusion:**

We strongly recommend the adoption of methanol fixation for sample collection strategies in new clinical trials. This approach is immediately available, is logistically simple and can offer cheaper and more reliable mutation calling than traditional NBF fixation.

## Abstract (precis)

High-quality tumour DNA is essential for any personalised treatment strategy based on NGS. Here we show that methanol fixation is superior to formalin, greater DNA yield, longer fragment size and more accurate copy-number calling using sWGS. We also show provide a new approach to understand fixation artefacts using non-negative matrix factorization.

## introduction

Although microscopic examination of formalin-fixed paraffin-embedded (FFPE) material remains crucial in cancer diagnosis, next-generation sequencing (NGS) of tumour DNA has emerged as a powerful diagnostic tool [1] and is a central component of personalised medicine initiatives. NGS relies heavily on high-quality DNA, and snap-frozen (SF) samples are preferred because formalin fixation induces chemical modifications and degradation of DNA [2, 3].

Comprehensive diagnostic strategies and translational research protocols therefore currently demand two samples, one SF for molecular analysis and the other FFPE for routine haematoxylin and eosin staining (H&E) and immunohistochemistry (IHC). Processing of SF samples for NGS has several disadvantages, including reduced ability to microdissect tumour material and significantly increased costs [4, 5]. In particular, there are significant barriers to obtaining SF material in large-scale clinical trials, where samples are typically collected from multiple hospitals in different countries. Therefore, alternatives to formalin-based fixation are required to circumvent the need for fresh-frozen sampling.

Methanol-based fixation has emerged as a promising such alternative [5–7] (supplementary Table S5, available at *Annals of Oncology* online). Universal molecular fixative (UMFIX) has been shown to be superior for IHC to neutral-buffered formalin (NBF), and gives higher yield and molecular weight of extracted DNA and RNA [5, 6, 8]. In addition, prolonged exposure to methanol fixatives may have fewer deleterious effects on DNA/RNA quantity and quality than NBF [3, 5]. However, potential NGS sequencing artefacts from methanol fixation have not been studied.

Here, we have tested the suitability of DNA extracted after methanol-based fixation for NGS assays compared with DNA from matched NBF and fresh-frozen tissues. We studied high-grade serous ovarian cancer (HGSOC) samples because they have ubiquitous *TP53* mutation and *TP53* sequences have been extensively studied for fixation artefacts [9, 10]. HGSOC also has marked genomic rearrangement and copy-number abnormalities (CNAs), which allow stringent inspection of the effects of DNA fragment length size on CNA profiling.

## patients and methods

### sample acquisition and processing

Three equal fragments were macrodissected from tumour specimens removed from 16 patients, median age 62, with HGSOC undergoing debulking surgery. In addition, mock biopsies of the tumour were taken from 12 cases with a 16G core biopsy gun. All samples were reviewed by at least two pathologists and fixed in 10% NBF (Genta Medical, York, UK)), UMFIX (Sakura Finetek, Thatcham, UK) or SF (liquid nitrogen). Matched normal tissue controls were processed in parallel. Full clinical details are given in supplementary Table S1, available at *Annals of Oncology* online.

### immunohistochemistry

5 µm sections of NBF and UMFIX fixed material were stained for CK7, p53, PAX8, WT1 and CK20 using established clinical protocols in the Department of Pathology, Queen Elizabeth University Hospital, Glasgow, with additional optimization for WT1 staining of UMFIX tissues. Staining and image analysis protocols, as well as all histoscore data, are described in supplementary material, available at *Annals of Oncology* online.

### DNA extraction and quantification

DNA was extracted using QIAmp DNA Micro and AllPrep DNA/RNA Micro Kit for UMFIX/NBF-fixed and SF tumours, respectively. DNA size distribution and quality were assessed by qPCR with Illumina FFPE QC Kit and KAPA hgDNA Quantification and QC Kit, respectively.

### tagged-amplicon sequencing (TAm-seq)

The coding regions of *TP53*, *PTEN*, *EGFR*, *PIK3CA*, *KRAS* and *BRAF* were sequenced by TAm-Seq as described previously [11] on an Illumina MiSeq using PE-125 bp protocols. Data analysis is described in supplementary material, available at *Annals of Oncology* online.

### shallow whole-genome sequencing (sWGS)

WGS libraries were prepared from 100 ng DNA using modified TruSeq Nano DNA LT Sample Prep Kit protocol. Library quality and quantity were assessed with DNA-7500 kit on 2100 Bioanalyzer and with Kapa Library Quantification kit according to the original protocols, respectively. Eighteen barcoded libraries were pooled together in equimolar amounts and each pool was sequenced on HiSeq2500 in SE-50 bp mode. Analysis methods are described in supplementary material, available at *Annals of Oncology* online.

### mutation signature analysis

Non-negative matrix factorisation was carried out to identify mutation signatures [12] in relation to different fixation (supplementary material, available at *Annals of Oncology* online). All non-reference base changes observed across the sequencing data were interrogated from both TAm-Seq and sWGS data.

## results

**Figure 1.**
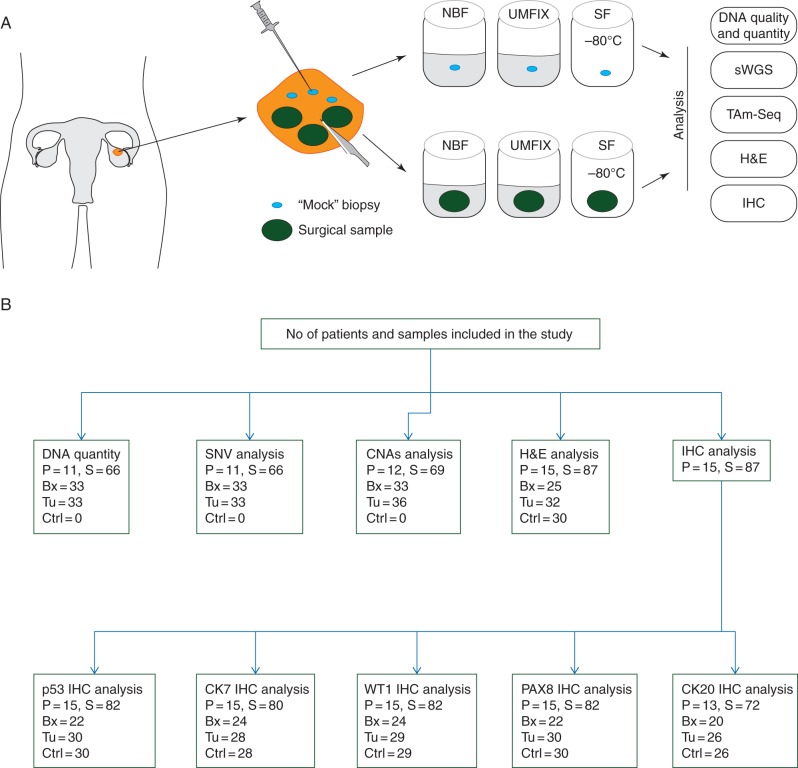
Study design. (A) Operative specimens from women undergoing surgery for HGSOC were sampled with a scalpel to acquire three surgical tumour samples and a 16G needle was used to obtain three mock biopsies. Matched surgical and biopsy samples from each case, with matched control tissue, were processed in parallel with fixation in NBF or UMFIX, or SF before downstream analysis. (B) Sample workflow: numbers of patients (P) and samples (S) used for analysis. Bx, biopsy; Tu, surgical tumour fragment; Ctrl, control tissue.

Figure 1 summarises the study design and the flow of samples through the study. Additional REMARK data are provided in supplementary material, available at *Annals of Oncology* online.

### methanol fixation yields higher yield and size of DNA fragments than buffered formalin

**Figure 2.**
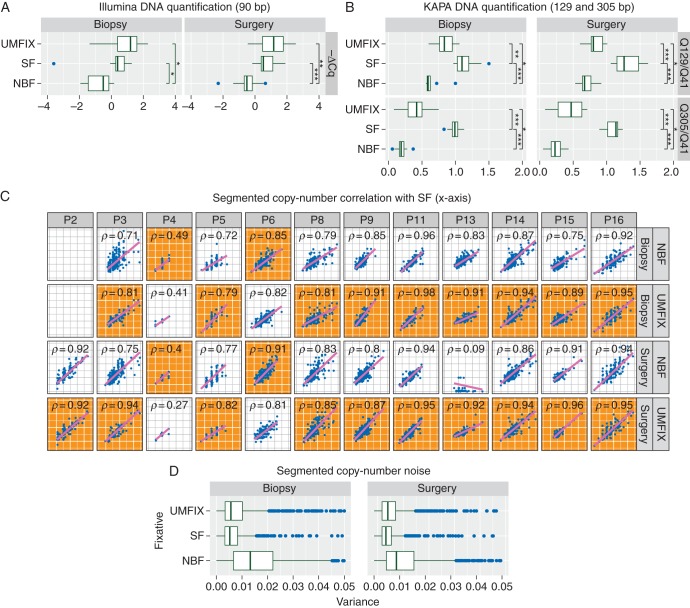
DNA yield and copy-number calling performance. Box plots show results of PCR assays for DNA size after extraction from SF, NBF and methanol (UMFIX) fixation from matched biopsy and surgical samples from 11 HGSOC patients. (A) Observed ΔCq values for DNA yield of 90 bp fragments (negative ΔCq values are shown for convenience). *P < 0.05, **P < 0.005, ***P < 0.0005. (B) Observed Q ratios for 129 bp/41 bp (top) and 305 bp/41 bp (bottom) fragments. Vertical brackets indicate Wilcoxon rank-sum test for difference in means: *P < 0.05, **P < 0.005, ***P < 0.0005. (C) Scatter plots show correlation between median normalised copy-number profiles from shallow WGS of SF compared with NBF or UMFIX biopsy and surgical samples from 12 patients. Spearman's rank-sum correlation rho is shown. Gray background (orange online) indicates plots with the highest correlation between UMFIX and NBF for each patient sample (biopsy or surgery). (D) Boxplots show an observed variance for each copy-number segment (*n* = 90 312) in 69 samples from 12 patients.

There was no significant difference in tumour cellularity and *TP53* allele fraction between UMFIX and NBF samples, thus allowing a direct comparison of DNA metrics (supplementary Figure S1, available at *Annals of Oncology* online). Quantification of extracted DNA showed similar yields of small (90 bp) fragments from UMFIX and SF samples, both of which were significantly higher than from NBF (Figure 2A). As expected, SF samples showed the highest yields of large fragments (129 bp, 305 bp), but yields from UMFIX samples were still significantly higher than NBF (Figure 2B).

### copy-number calling in methanol-fixed material is superior to formalin

Copy-number profiles from sWGS were compared for correlation and variance of copy-number abnormality (CNA) estimation, using SF as gold standard. UMFIX showed superior copy-number profiles compared with NBF, with 9 of 11 biopsies and 10 of 12 surgical samples showing higher correlation with the matched SF (Figure 2C). UMFIX also had lower noise for segmental copy-number estimation than NBF (Figure 2D).

### single-nucleotide sequencing noise from methanol-fixed material is comparable with SF and NBF

**Figure 3.**
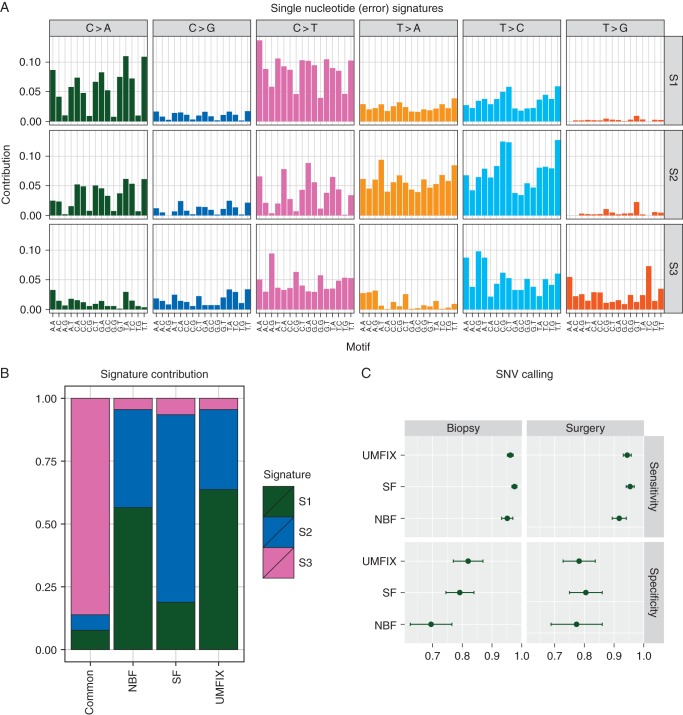
Single-nucleotide noise profiles and variant calling performance. (A) Bar plots of the three somatic mutation signatures (S1–S3) identified by non-negative matrix factorisation using all non-reference bases observed in sWGS and TAm-SEQ sequencing data in 69 samples from 12 patients (*n* = 255 376). Bar plots are grouped by the observed base change with individual bars showing the proportion observed at different trinucleotide sequences. (B) Stacked bar plots show the proportion of the three mutation signatures observed only in SF and NBF or UMFIX fixed samples compared with signatures present in all samples from an individual patient (common). (C) Sensitivity (top) and specificity (bottom) for manually curated SNV calls (*n* = 546) from TAm-SEQ of biopsy and surgical samples from 11 patients. Bars indicate the 95% confidence interval around the indicted mean.

We analysed low-level sequence noise using 255 376 observed non-reference bases in the sWGS and TAm-Seq data. All analysed mutations were filtered using dbSNP specifically to exclude germline SNPs. Analysis of the flanking bases around each base change revealed three mutation signatures (Figure 3A): signature 1 was dominated by non-CpG C>A transversions and C>T transitions; Signature 2 had high rates of T>A, C>A, T>C and C>T transitions, with the latter enriched in the trinucleotide context NCA (where N indicates any base); Signature 3 showed T>C and CpG-related C>T transitions. A breakdown of the contribution of each signature across four categories (base changes common to all samples, changes unique to SF, UMFIX and NBF) showed that the common changes (containing a collection of both true SNVs and typical errors) were dominated by Signature 3, whereas the other categories were a mix of Signatures 1 and 2 (Figure 3B).

### single-nucleotide variant calling from methanol-fixed material is comparable with fresh-frozen

SNVs were called using TAm-Seq of 66 samples yielding 546 variants. Manual curation of these variants revealed lower average sensitivity and specificity for NBF compared with SF and UMFIX, albeit not significantly (Figure 3C).

### methanol fixation permits high-quality H&E and IHC analyses

**Figure 4.**
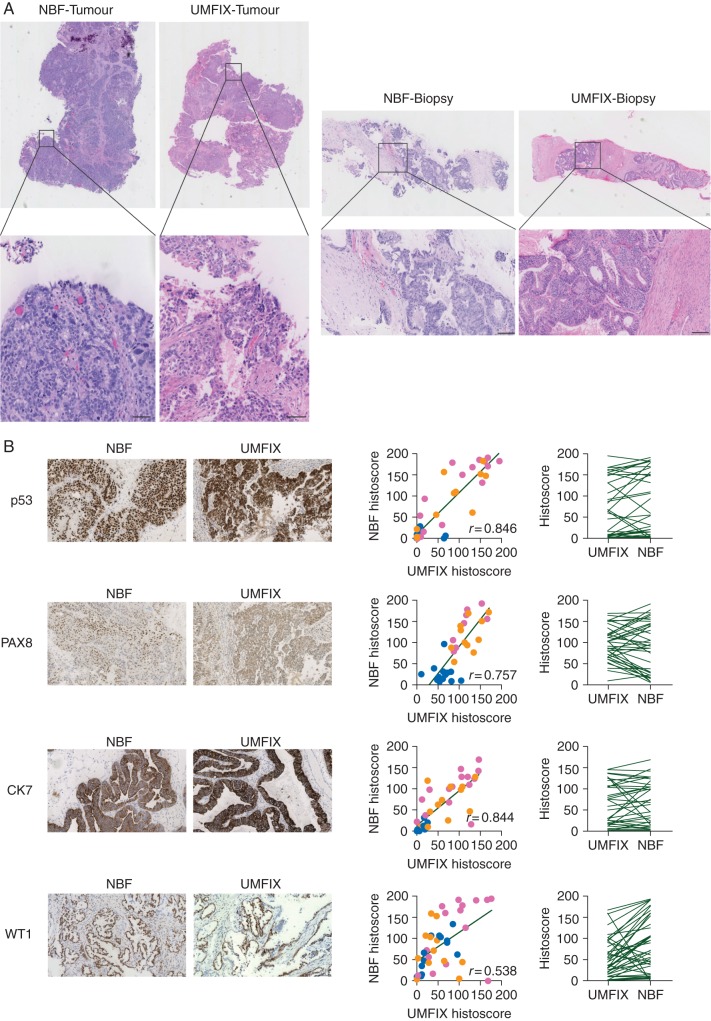
H&E staining and IHC scoring. (A) H&E staining of tumour fragments (left) and biopsies (right) from matched tissues fixed in NBF and UMFIX. Bars represent 100 µm. (B) Representative images (left) show IHC staining for p53, PAX8, CK7, WT1 on matched NBF and UMFIX tissues. Quantitative histoscores (middle) of intensity of staining for each IHC marker on tumour fragments [dark gray points (pink online)], biopsies [light gray points (orange online)] and control tissue [black points (blue online)] samples. Spearman's rank-sum correlation rho is shown (*P* < 0.001 for all analyses). Paired data plots (right) show comparison of median histoscores from paired UMF- and NBF-fixed tissues for each IHC marker. Median scores were only significantly different for WT staining (*P* = 0.011).

Tissue morphology (H&E staining) of UMFIX samples was comparable with NBF fixation. Overall, differences between UMFIX and NBF were not diagnostically significant (Figure 4A). Statistically significant correlation was found between quantitative IHC histoscores in UMFIX and NBF-fixed samples for key HGSOC markers (p53, CK7, PAX8, WT1). CK20 was uniformly negative in all tumour samples, regardless of fixative (data not shown). There was no significant difference in median histoscore between the two sample sets for p53, CK7 and PAX8 (Figure 4B).

## discussion

The most important variables for NGS assays are DNA quality and yield. Formalin fixation can induce severe effects on the structure and integrity of DNA causing C>T, A>G, G>T, G>C and A>T base changes, methylene bridge formation, DNA denaturation and DNA fragmentation [6, 13–15]. After NGS, these chemical modifications result in greater SNV artefacts, higher sequence duplication rates, smaller insert sizes and lower fractions of mappable reads [16, 17].

We evaluated whether methanol-based fixation can reduce these detrimental artefacts when attempting to identify true somatic SNVs and accurate copy-number from clinical material. We show that UMFIX fixation yields longer amplifiable DNA fragments, in agreement with previous reports [3, 5, 8], which improves our ability to call DNA copy-number accurately. We show that SNV calling from UMFIX DNA has similar performance to DNA from SF tissues and that traditional H&E staining and IHC scoring can be carried out on UMFIX-embedded samples with minimal optimisation.

These findings are clinically highly important: although attempts have been made to reduce noise induced by formalin fixation (e.g. increasing targeted sequencing coverage or reducing C>T transitions with UDG treatment), these methods only mitigate some sources of noise when calling SNVs and do not improve the ability to call CNA [18, 19]. CNA detection is more challenging than SNV detection and remains the major clinical need for personalised treatment approaches in HGSOC.

In addition, we have used a state-of-the-art computational approach to perform in-depth exploration of the low-level sequence noise introduced by fixation and sample processing. In an advance over previous approaches, we modelled the trinucleotide context of each base change and de-convolved distinct trinucleotide noise signatures. This computational approach has previously been used to identify signatures in collections of SNVs observed across thousands of tumours, and these signatures used to infer underlying mutational processes [12]. In our data, we identified three distinct trinucleotide signatures. Signature 3 has high similarity to a previously identified CpG-age-related cytosine deamination (C>T) signature (signature 1B [12]), and a recently uncovered sequence error signature [20]. However, signatures 1 and 2 are novel and have no similarity to previously described signatures. In particular, they show high rates of C>T transition but not in CpG dinucleotide contexts. As expected, signature 3 contributed only to the set of base changes common to all samples across a patient. In contrast, signatures 1 and 2 contributed only to the base changes exclusive to SF, UMFIX or NBF samples. This suggests that the sequencing noise represented by these two signatures (C>T not at CpG) is induced through sample processing. The two fixative conditions showed a slightly increased contribution to signature 1 compared with SF, suggesting that fixation may have a specific effect. However, larger studies are required to achieve the power to discern this. This approach to modelling sequence noise provides powerful tools to explore sequencing artefacts and an analytical framework to understand the mechanisms behind their creation. Further studies with high coverage WGS are now underway to refine these data.

There are no data on the effects of long-term methanol fixation on DNA quality or quantity, and this study utilised samples collected no more than 6 months before analysis. With FFPE material, it is possible to isolate DNA from long-term archived samples [21], although factors such as duration of fixation, age of the sample, exposure to heat and light, as well as the concentration, buffering and age of the formalin, can all influence DNA quality and extent of sequence artefact [22]. Careful longitudinal analyses will be required to ascertain whether similar problems emerge in UMFIX samples.

We specifically did not examine RNA in this study. There are several previous publications on the utility of RNA extracted from methanol-fixed specimens in PCR and microarray assays, including from samples stored at room temperature for up to 8 weeks [5, 23]. However, we are not aware of any study assessing RNA sequencing or RNA profiling of samples extracted from methanol-fixed tissue—again, future studies will be required to confirm whether RNA extracted from methanol can be reliably used in such assays.

In summary, whilst SF samples remain the gold standard for nucleic acid extraction from tumour material at present, there are significant costs associated with such samples in clinical trials and NGS-based personalised medicine studies. A key advantage of methanol fixation is that it allows easy collection and embedding of tumour material with associated economies for pathological verification and microdissection. Based on our findings of superior DNA quality, we recommend that UMFIX be routinely adopted for collection and storage of clinical cancer specimens for large-scale genomic analysis.

## Supplementary Material

Supplementary Data
