# Supplementary material for: Methanol-based fixation is superior to buffered formalin for next-generation sequencing of DNA from clinical cancer samples
Source: Ann Oncol. 2015 Dec 17;27(3):532–9. doi: 10.1093/annonc/mdv613 (PMC4769995; doi:10.1093/annonc/mdv613)
Supplement: Supplementary Data [file supp_27_3_532__index.html]

Methanol-based fixation is superior to buffered formalin for next-generation sequencing of DNA from clinical cancer samples — Methanol-based fixation is superior to buffered formalin for next-generation sequencing of DNA from clinical cancer samples — Methanol-based fixation is superior to buffered formalin for next-generation sequencing of DNA from clinical cancer samples — Supplementary Data 

# Methanol-based fixation is superior to buffered formalin for next-generation sequencing of DNA from clinical cancer samples

## Supplementary Data

Supplementary Data

- Supplementary Data - Pdf file
