# Supplementary material for: Methanol-based fixation is superior to buffered formalin for next-generation sequencing of DNA from clinical cancer samples
Source: Ann Oncol. 2015 Dec 17;27(3):532–9. doi: 10.1093/annonc/mdv613 (PMC4769995; doi:10.1093/annonc/mdv613)
Supplement: Supplementary Data [file supp_mdv613_mdv613supp.pdf]

# Supplementary Material:

## Methanol-based fixation is superior to buffered formalin for next-generation sequencing of clinical cancer samples

Anna M. Piskorz*, Darren Ennis*, Geoff Macintyre*,
Teodora E. Goranova, Matthew Eldridge, Nuria Segui-Gracia, Mikel Valganon,
Aoisha Hoyle, Clare Orange, Luiza Moore, Mercedes Jimenez-Linan, David Millan,
Iain A. McNeish#, James D. Brenton#

This supplementary document has been generated using *sweave*. The document has an associated sourcecode file which contains a mix of latex and R which when compiled generates the pdf. Each figure in this document is generated dynamically during compilation. For clarity, the code which generates each of these plots is hidden. This code can be accessed by looking at the sourcecode which can be found in the following repository https://bitbucket.org/britroc/fixation. A description of how to reproduce the entire analysis associated with the manuscript can be found at the end of this document. Patient sequencing data can be accessed via application through the European Genome Archive, accesssion EGAS00001001433.

## 1 Samples

Tissue samples were collected from 16 women (median age 62) with HGSOC undergoing debulking surgery at Glasgow Royal Infirmary, Glasgow, UK. 7/16 samples were acquired at primary debulking surgery, the remaining 9 following 3 - 4 cycles of carboplatin and paclitaxel chemotherapy (summarised in Supplementary Table 1). All samples were collected under the auspices of NHS Greater Glasgow and Clyde Biorepository following approval by West of Scotland Research Ethics Committee 4 (Reference 10/S0704/60). All samples were reviewed by at least two pathologists. Small samples (approximately $20mm^3$) of both tumour and normal tissue were macrodissected, divided into three equal fragments, and fixed in 10% neutral-buffered formalin (Genta Medical, York, UK), UMFIX (Sakura Finetek, Thatcham, UK) or snap frozen (liquid nitrogen). In addition, separate three or more 16G core biopsies (Argon Medical Devices, Athens, TX, USA) of the main tumour mass were taken from 12 cases. After 24 hours, UMFIX and NBF-fixed samples were processed in a Thermo Excelsior Tissue Processor using optimised protocols (Supplementary Table 2 and 3). NBF/UMFIX-fixed normal sample pairs (tumour cells less than 10%) were used only for IHC analysis as controls. A summary of all sample details can be found in Supplementary Table 7.

**Supplementary Table 1:** Clinical patient information: This table summarises relevant clincial information for the 16 patients included in this study. * - no biopsy cores, IPS - Immediate primary surgery, DPS - Delayed primary surger

| Patient ID | Age | Debulking status | Malignant Tissue Type | Normal Tissue | Stage |
|---|---|---|---|---|---|
| P5 | 48 | IPS | Ovary | Fallopian Tube | 3C |
| P3 | 56 | IPS | Ovary | Fallopian Tube | 3C |
| P16 | 69 | IPS | Ovary | Fallopian Tube | 3A2 |
| P13 | 82 | DPS | Ovary | Uterus | 3C |
| P9 | 57 | IPS | Ovary | Cervix | 2A |
| P11 | 71 | DPS | Omental deposit | Fallopian Tube | 3C |
| P6 | 53 | DPS | Ovary | Myometrium | 3C |
| P15 | 68 | DPS | Omental deposit | Fallopian Tube | 3C |
| P14 | 57 | DPS | Ovary | Uterus | 3C |
| P8 | 72 | IPS | Ovary | Uterus | 3A |
| P4 | 48 | DPS | Ovary | Uterus | 4A |
| P12 | 65 | DPS | Omental deposit | Terminal ileum | 4A |
| P7* | 71 | IPS | Ovary | Uterus | 3C |
| P10* | 68 | DPS | Ovary | Myometrium | 4B |
| P2* | 56 | IPS | Ovary | Uterus | 3C |
| P1* | 58 | DPS | Vaginal lump | Myometrium | 3C |

**Supplementary Table 2:** UMFIX processing cycle: This table contains experimental details for universal molecular fixative processing protocol carried out in this study.

| Reagent | Time (mins) | Temperature | Drain | Vacuum | Agitation |
|---|---|---|---|---|---|
| 70% Ethanol | 35 | $32/40\,^{\circ}$C | 60 | Yes | 5 |
| 90% Ethanol | 40 | $32/40\,^{\circ}$C | 60 | Yes | 5 |
| 95% Ethanol | 45 | $32/40\,^{\circ}$C | 60 | Yes | 5 |
| 100% Ethanol | 50 | $32/40\,^{\circ}$C | 60 | Yes | 5 |
| 100% Ethanol | 50 | $32/40\,^{\circ}$C | 60 | Yes | 5 |
| 100% Ethanol | 55 | $32/40\,^{\circ}$C | 60 | Yes | 5 |
| Xylene | 45 | $32/40\,^{\circ}$C | 60 | Yes | 5 |
| Xylene | 50 | $32/40\,^{\circ}$C | 60 | Yes | 5 |
| Xylene | 60 | $32/40\,^{\circ}$C | 120 | Yes | 5 |
| Wax | 70 | $61\,^{\circ}$C | 120 | Yes | 5 |
| Wax | 80 | $61\,^{\circ}$C | 120 | Yes | 5 |
| Wax | 100 | $61\,^{\circ}$C | 120 | Yes | 5 |

**Supplementary Table 3:** NBF processing cycle: This table contains experimental details for neutral buffered formalin processing protocol carried out in this study.

| Reagent | Time (mins) | Temperature | Drain | Vacuum | Agitation |
|---|---|---|---|---|---|
| 10% NBF | 30 | $Amb/40\,°$C | 60 | Yes | 5 |
| 70% Ethanol | 35 | $32/40\,°$C | 60 | Yes | 5 |
| 90% Ethanol | 40 | $32/40\,°$C | 60 | Yes | 5 |
| 95% Ethanol | 45 | $32/40\,°$C | 60 | Yes | 5 |
| 100% Ethanol | 50 | $32/40\,°$C | 60 | Yes | 5 |
| 100% Ethanol | 50 | $32/40\,°$C | 60 | Yes | 5 |
| 100% Ethanol | 55 | $32/40\,°$C | 60 | Yes | 5 |
| Xylene | 45 | $32/40\,°$C | 60 | Yes | 5 |
| Xylene | 50 | $32/40\,°$C | 60 | Yes | 5 |
| Xylene | 60 | $32/40\,°$C | 120 | Yes | 5 |
| Wax | 70 | $61\,°$C | 120 | Yes | 5 |
| Wax | 80 | $61\,°$C | 120 | Yes | 5 |
| Wax | 100 | $61\,°$C | 120 | Yes | 5 |

# 2 Immunohistochemistry

Five $\mu$m sections were cut from available surgical samples, biopsies and control samples and were stained for Cytokeratin 7 (CK7), p53, PAX8, WT1 and CK20 on a fully automated Leica Bond III (Leica Microsystems (UK), Milton Keynes, UK) using established clinical protocols in the Department of Pathology, Queen Elizabeth University Hospital Glasgow, with appropriate positive and negative controls. Staining for WT1 on UMFIX tissue was subsequently optimised on a Dako Autostainer (CRUK Beatson Institute, Glasgow, UK). For WT1, antigen retrieval ($98\,°$C, 45 minutes, citrate buffer (pH 9)) was followed by 5 minutes endogenous peroxidase block (3% H2O2). Primary antibody was added for 60 minutes at $25\,°$C. Antibody binding was visualized using DAB (3-39 diaminobenzidine). Details of antibodies used and dilutions are found in Supplementary Table 4.

Stained slides were digitised (Hamamatsu NanoZoomer NDP, Hamamatsu Photonics, Welwyn Garden City, UK) and viewed using Slidepath Digital Image Hub V4.0.7 (Leica Microsystems, Milton Keynes). Areas of tumour were identified and scored using Slidepath Tissue Image Analysis and histoscores generated by multiplying intensity of cellular staining within marked areas (range 0-3) by percentage cells with positive staining (range 0-100), with a maximum histoscore of 300. Data were analysed using GraphPad Prism version 6.0 (GraphPad software, San Diego, California, USA) - Spearman rho was used to correlate UMFIX and NBF histoscores, and paired t-test used for pairwise histoscore comparisons. All analyses were two-tailed.

**Supplementary Table 4:** Immunohistochemical assays conditions: This table contains details on the antibodies and systems used for IHC.

| Antibody | Clone | Dilution | Manufacturer | Product Number |
|---|---|---|---|---|
| p53 | D0-7 | 1:300 | Leica Biosystems | PA0057 |
| WT1 | WT49 | 1:30 | Leica Biosystems | NCL-L-WT1-562 |
| CK7 | RN7 | 1:200 | Leica Biosystems | CK7-OVTL-R-7-CE |
| Pax8 | PAX8R1 | 1:40 | Abcam | Ab53490 |
| CK20 | PW31 | 1:100 | Leica Biosystems | PA0022 |

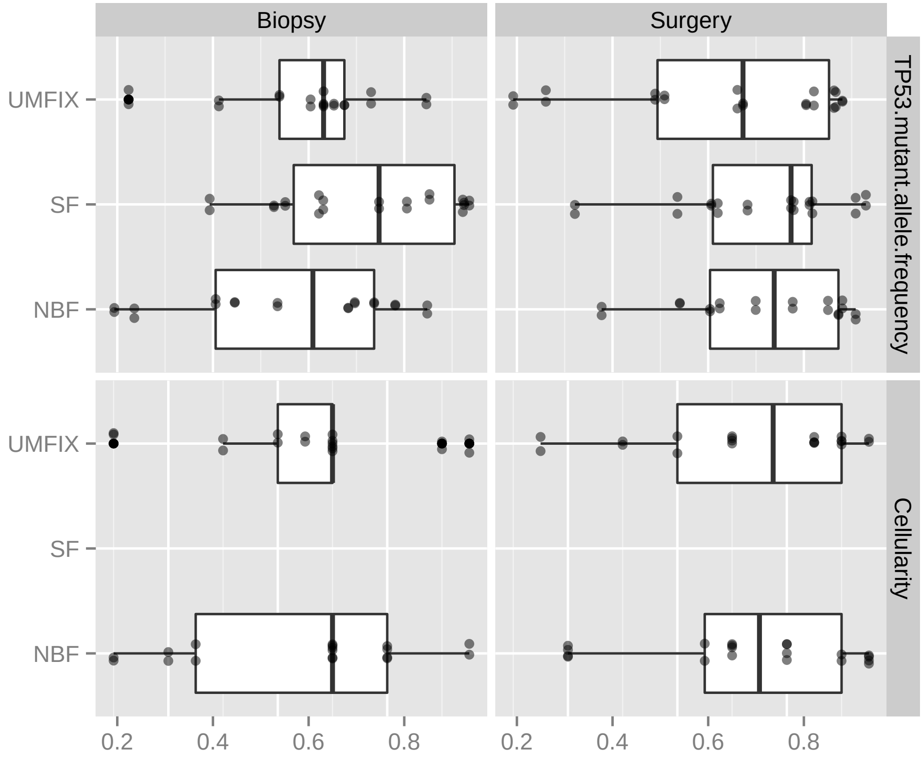


**Supplementary Figure 1:** TP53 mutant allele frequency and pathology estimated tumour cellularity: Boxplots show the distribution of weighted average TP53 mutant allele frequencies generated across TAm-Seq replicates (66 samples, 11 patients, top) and pathology estimated tumour cellularities (66 samples, 11 patients, bottom)

## 3 Pathology

Tumour cellularity was assessed by pathologist based on H&E staining performed for all fixed (UMFIX, NBF) biopsy and surgical samples (see Supplementary Table 6 and Supplementary Figure 1). The cellularity was determined as a percentage of tumor cells occupied selected for microdissection area. The microdissected areas were subjected to DNA extraction. Additionally the tumour cellularity was assessed on molecular level using TP53 mutant frequency calculated using TAmSeq data as a proxy.

## 4 DNA extraction

DNA was extracted from 10-60 sections ($10\mu$m thickness) of UMFIX/NBF-fixed tissues using QIAmp DNA Micro kit (Qiagen) with the following modification to the original protocol: an additional incubation step with Buffer ATL at $95\,^{\circ}$C for 15 minutes was introduced before adding proteinase K. Paraffin was removed by the standard xylene/ethanol method. DNA from SF tissues was extracted using AllPrep DNA/RNA Micro Kit (Qiagen) according to the manufacturer's recommendations.

## 5 DNA quantification and quality assessment

DNA quantification of all samples was performed by Qubit 2.0 Fluorometer (Life Technologies) using dsDNA BR Assay. DNA size distributions were analysed on the Agilent 2100 Bioanalyzer (Agilent Technologies) using the DNA 12000 Kit. All DNA samples were analysed in triplicate and all reactions were performed using the supplier's protocols.

### 5.1 qPCR of 90bp fragments using Illumina FFPE QC Kit (Illumina)

Results for quantification of 90bp fragments are summarised in Supplementary Figure 2. According to Illumina's recommendations, samples analysed by Illumina FFPE QC Kit that show $\Delta Cq \leq 2.0$ should be suitable for NGS preparation. All but two samples were above this threshold.
*Statistical test:* The Wilcoxon rank-sum test for difference between means was applied to the quantification of 90bp fragments for different conditions (plotted in Supplementary Figure 2). The resulting p-values, corrected for multiple testing using the Bonferroni method, are summarised below:

| | UMFIX vs SF | NBF vs SF | NBF vs UMFIX |
|---|---|---|---|
| Biopsy | 0.02791461 | 6.539409e-05 | 7.599508e-05 |
| Surgery | 0.08424366 | 6.539409e-05 | 8.244320e-06 |

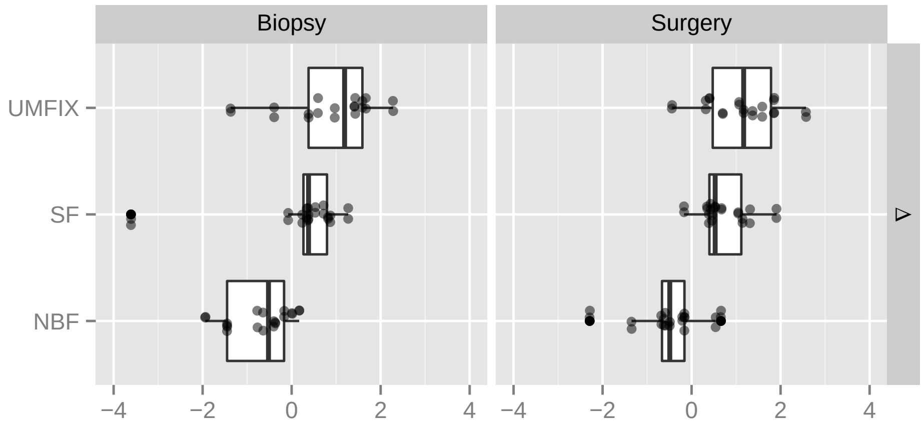


**Supplementary Figure 2:** Observed $-\Delta Cq$ (Figure 2a from paper): The boxplots show results of PCR assays for DNA size after extraction from snap frozen (SF), buffered formalin (NBF) and methanol (UMFIX) fixation from matched biopsy and surgical samples from 11 HGSOC patients. Negative $\Delta Cq$ values are shown for convenience.

### 5.2 qPCR of 41bp, 129bp and 305bp fragments using KAPA hgDNA Quantification and QC Kit (KAPA Biosystems)

Q-ratios (Q305/Q41 and Q129/Q41) were calculated for each sample after analysis with KAPA hgDNA Quantification and QC Kit and are summarised in Supplementary Figure 3.
*Statistical test:* The Wilcoxon rank-sum test for difference between means was applied to the quantification of Q-ratios for different conditions (plotted in Supplementary Figure 3). The resulting p-values, corrected for multiple testing using the Bonferroni method, are summarised below:

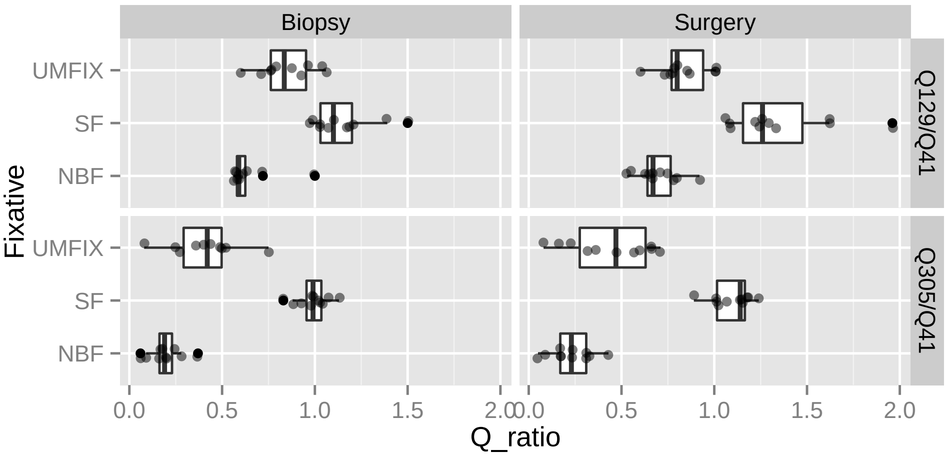


**Supplementary Figure 3:** Observed Q-ratios (Figure 2b from paper). The boxplots show results of PCR assays for DNA size after extraction from snap frozen (SF), buffered formalin (NBF) and methanol (UMFIX) fixation from matched biopsy and surgical samples from 11 HGSOC patients.

| | UMFIX vs SF | NBF vs SF | NBF vs UMFIX |
|---|---|---|---|
| Q305/Q41.Biopsy | 0.0009831970 | 0.0009831970 | 0.02178779 |
| Q305/Q41.Surgery | 0.0009424107 | 0.0009424107 | 0.03880384 |
| Q129/Q41.Biopsy | 0.0052738487 | 0.0012908224 | 0.01822342 |
| Q129/Q41.Surgery | 0.0009424107 | 0.0009424107 | 0.03880384 |

# 6 Shallow Whole Genome sequencing (sWGS)

DNA from each sample (100ng) was sheared on Covaris S220 (Covaris): duty cycle - 10%, intensity - 5.0, bursts per sec - 200, duration - 300 sec, mode - frequency sweeping, power - 23V, temperature - $5.5\,^{\circ}$C to $6\,^{\circ}$C, water level - 13. Libraries were prepared with the TruSeq Nano DNA LT Sample Prep Kit (Illumina) using a modified protocol - Sample Purification Beads were replaced by Agencourt AMPure XP beads (Beckman Coultier) and size selection after the End Repair was done to remove only the short fragments. Quality and quantity for contructed libraries were assessed with DNA 7500 kit on Agilent 2100 Bioanalyzer and with Kapa Quantification kit (KAPA Biosystems) on 7900HT Fast Real-Time PCR System (Applied Biosystems) according to the supplier's recommendations, respectively. Libraries from 18 barcoded samples were pooled together in equimolar amounts and each pool was loaded on a single lane of a HiSeq Single End Flowcell (Illumina), followed by cluster generation on a cBot (Illumina) and sequencing on a HiSeq 2500 (Illumina) in a single-read 50bp mode.

## 6.1 Read alignment and copy-number calling

Reads were aligned using bwa-mem v0.7.12-r1039 [10] to the 1000 genomes version of human genome build GRCh37. Picard (http://picard.sourceforge.net) was used to remove duplicate reads.
In order to perform legitimate comparisons between samples for copy-number calling, all samples must have approximately the same read coverage. Therefore, for each sample triplet (UMFIX, SF or NBF), reads were downsampled to the condition with the lowest number reads to account for any variation in

sequencing depth. QDNAseq [15] was used to filter reads then segment the genome and identify regions of copy-number change (see script copy_number_calling.R in the repository for details).

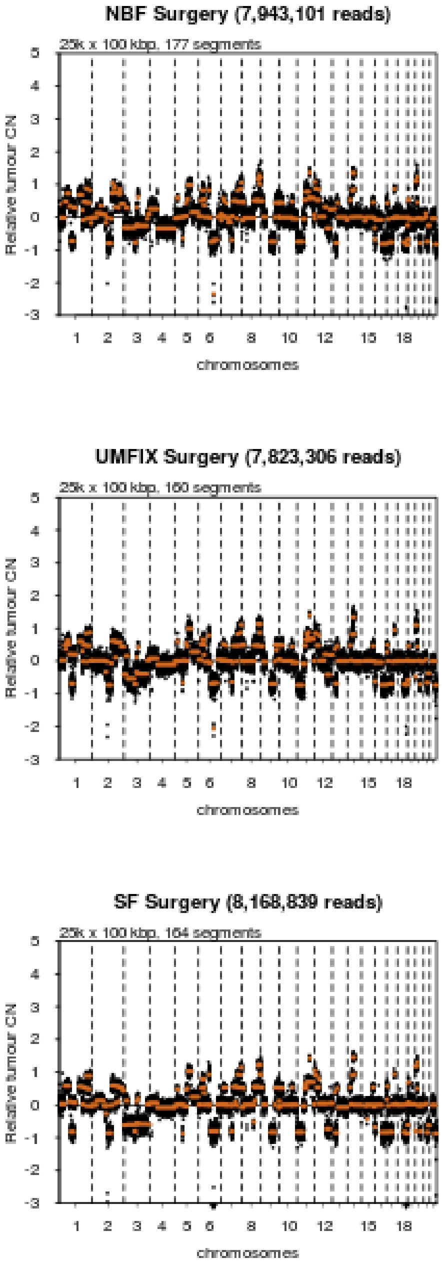


**Supplementary Figure 4:** Copy-number profiles for patient P2

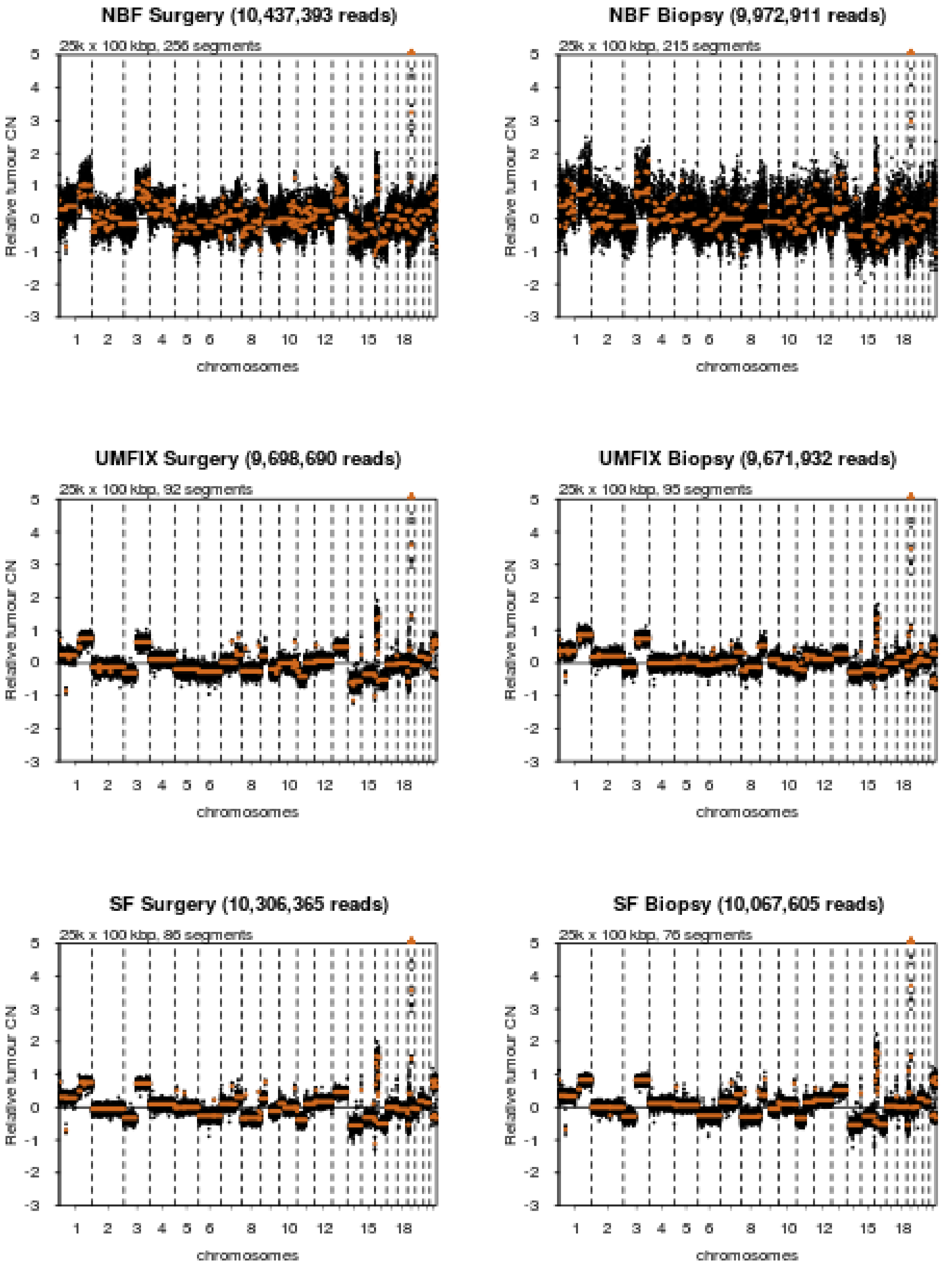


**Supplementary Figure 5:** Copy-number profiles for patient P3

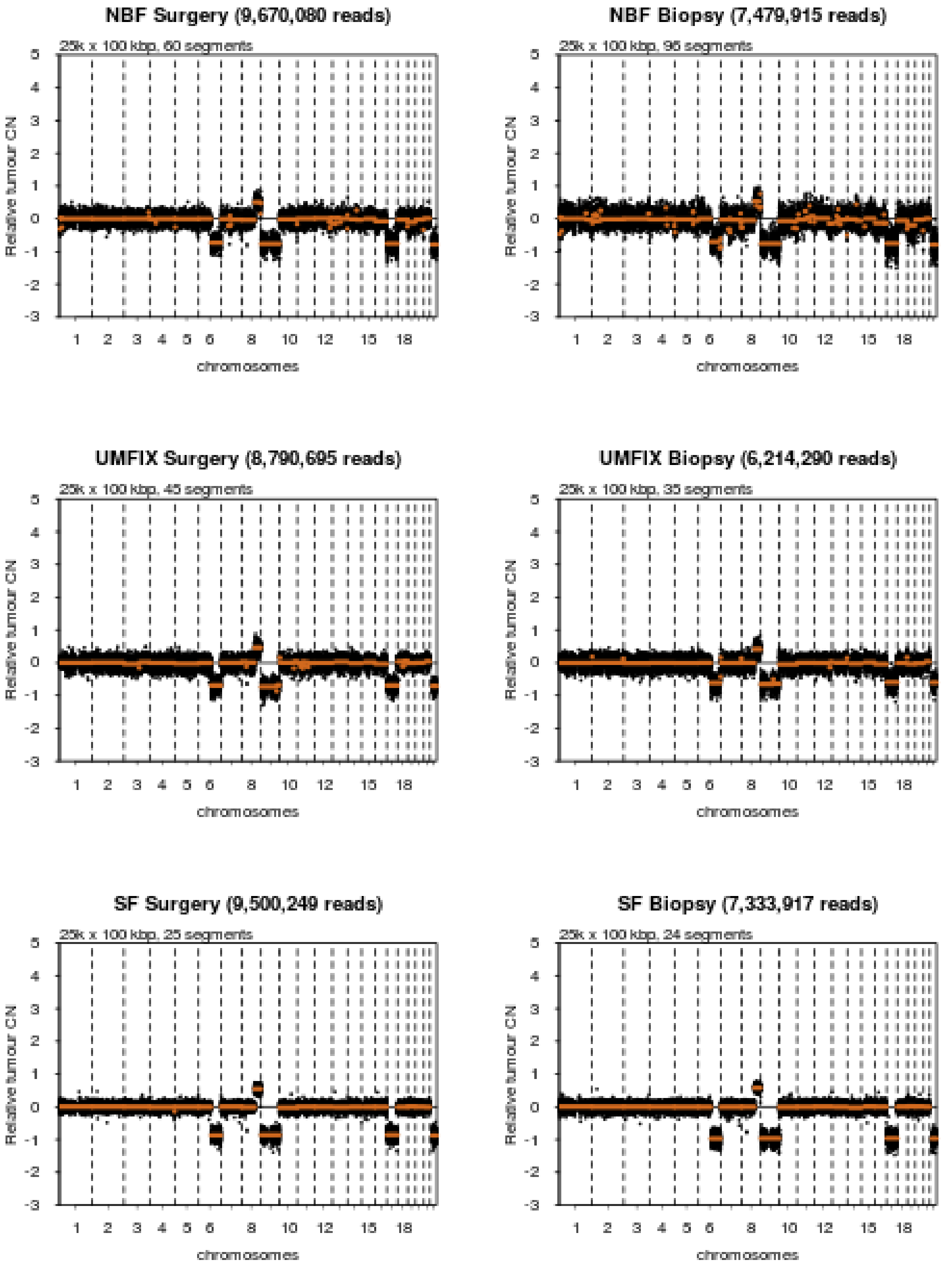


**Supplementary Figure 6:** Copy-number profiles for patient P4

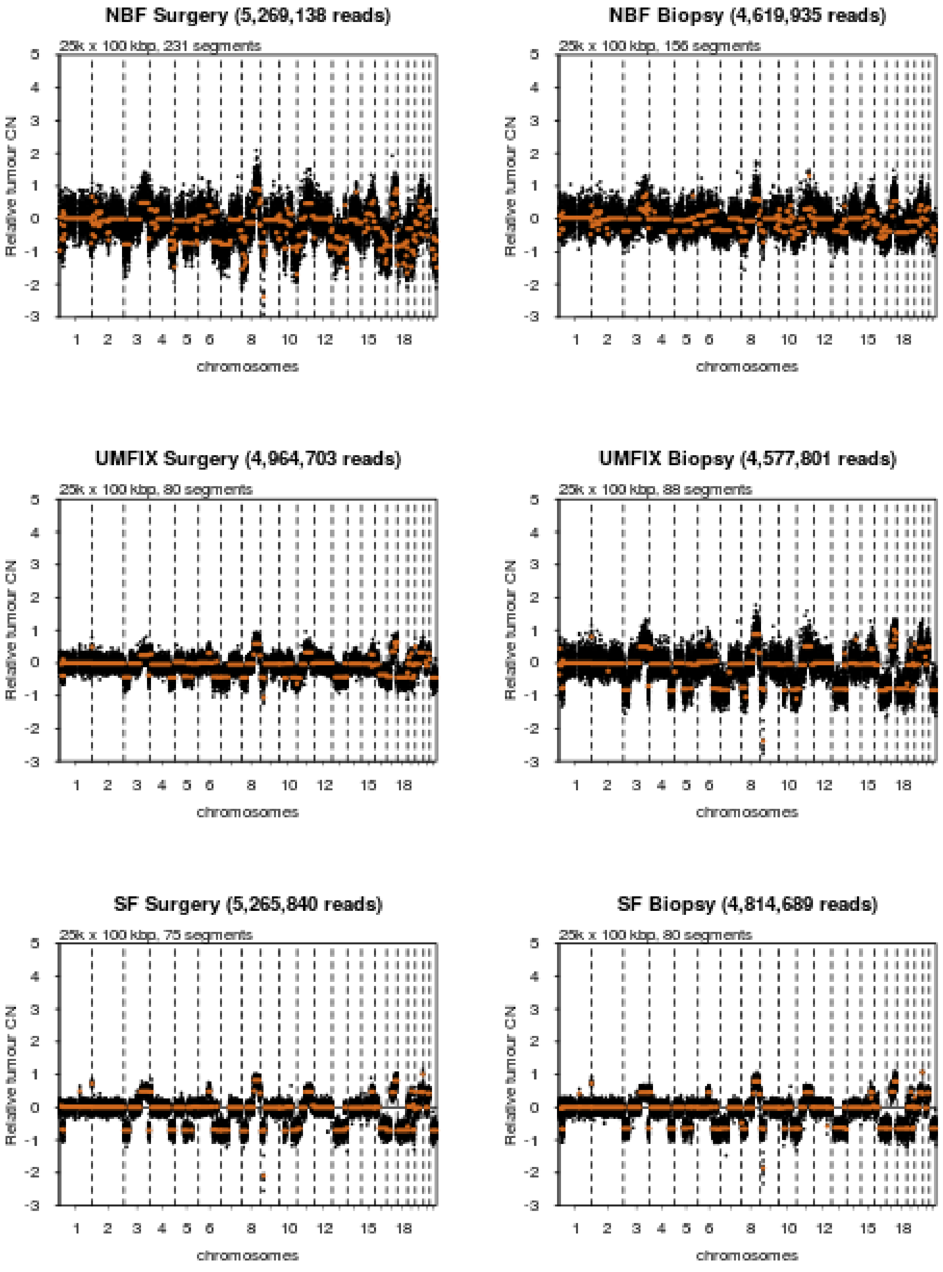


**Supplementary Figure 7:** Copy-number profiles for patient P5

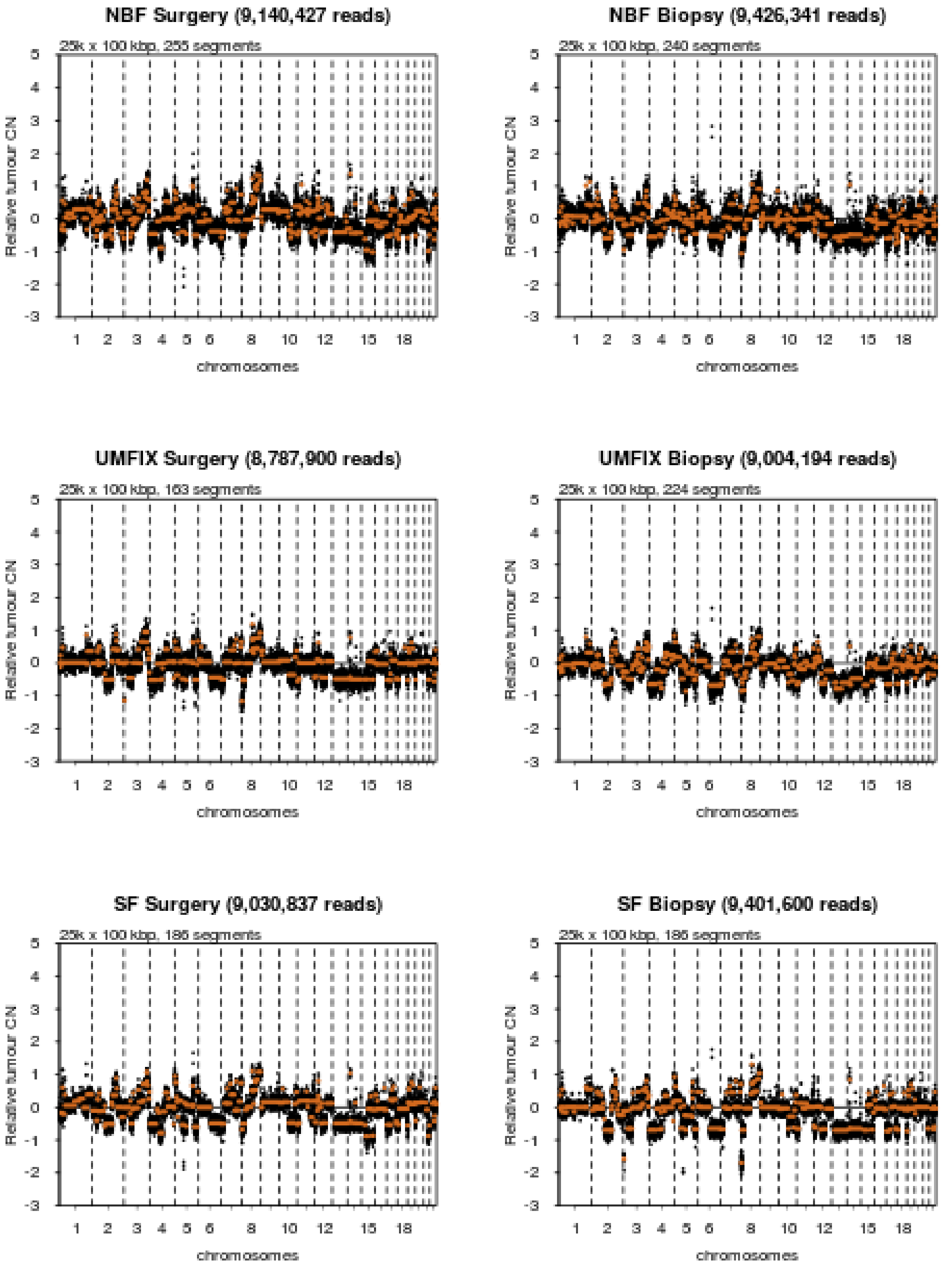


**Supplementary Figure 8:** Copy-number profiles for patient P6

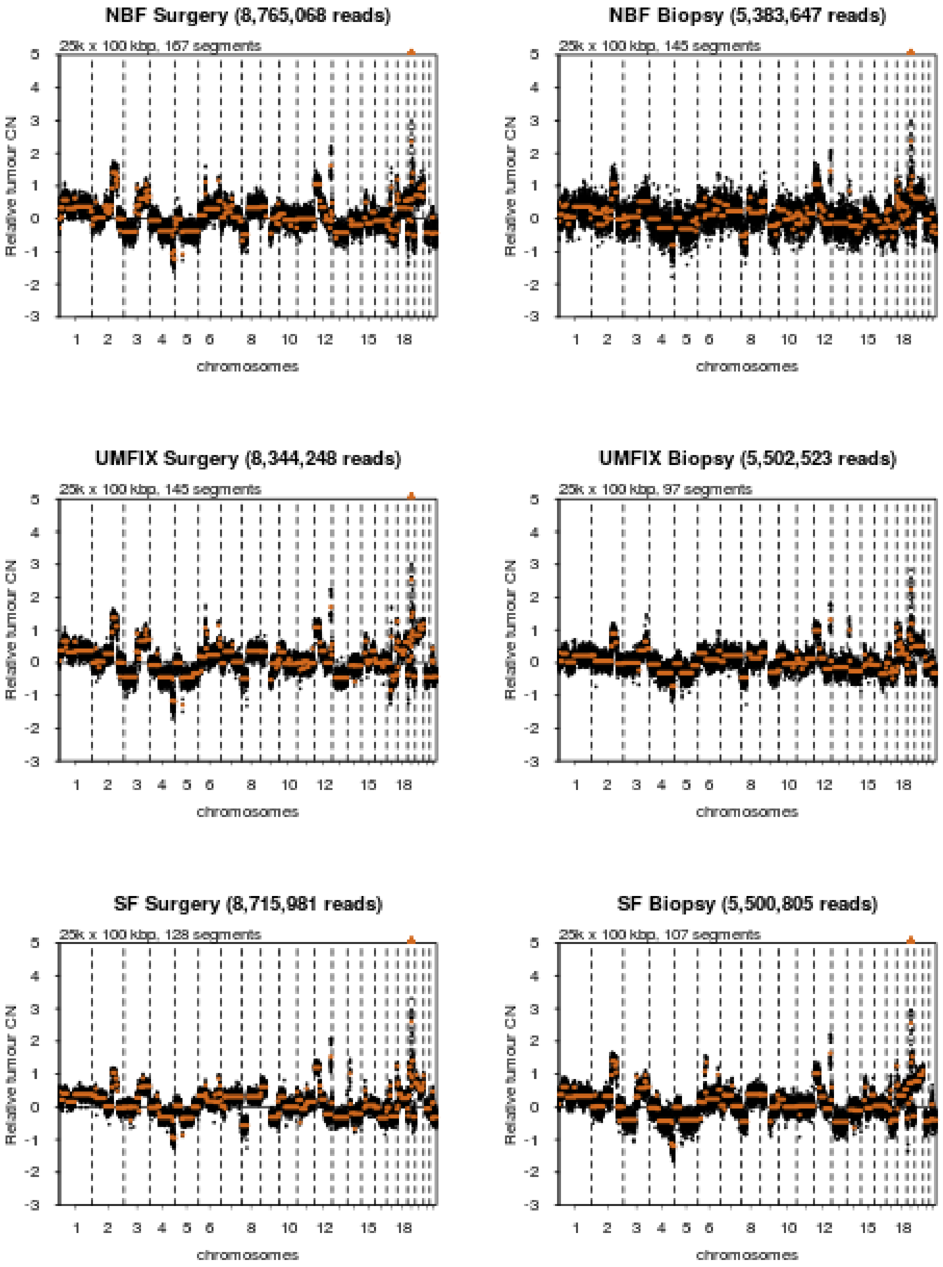


**Supplementary Figure 9:** Copy-number profiles for patient P8

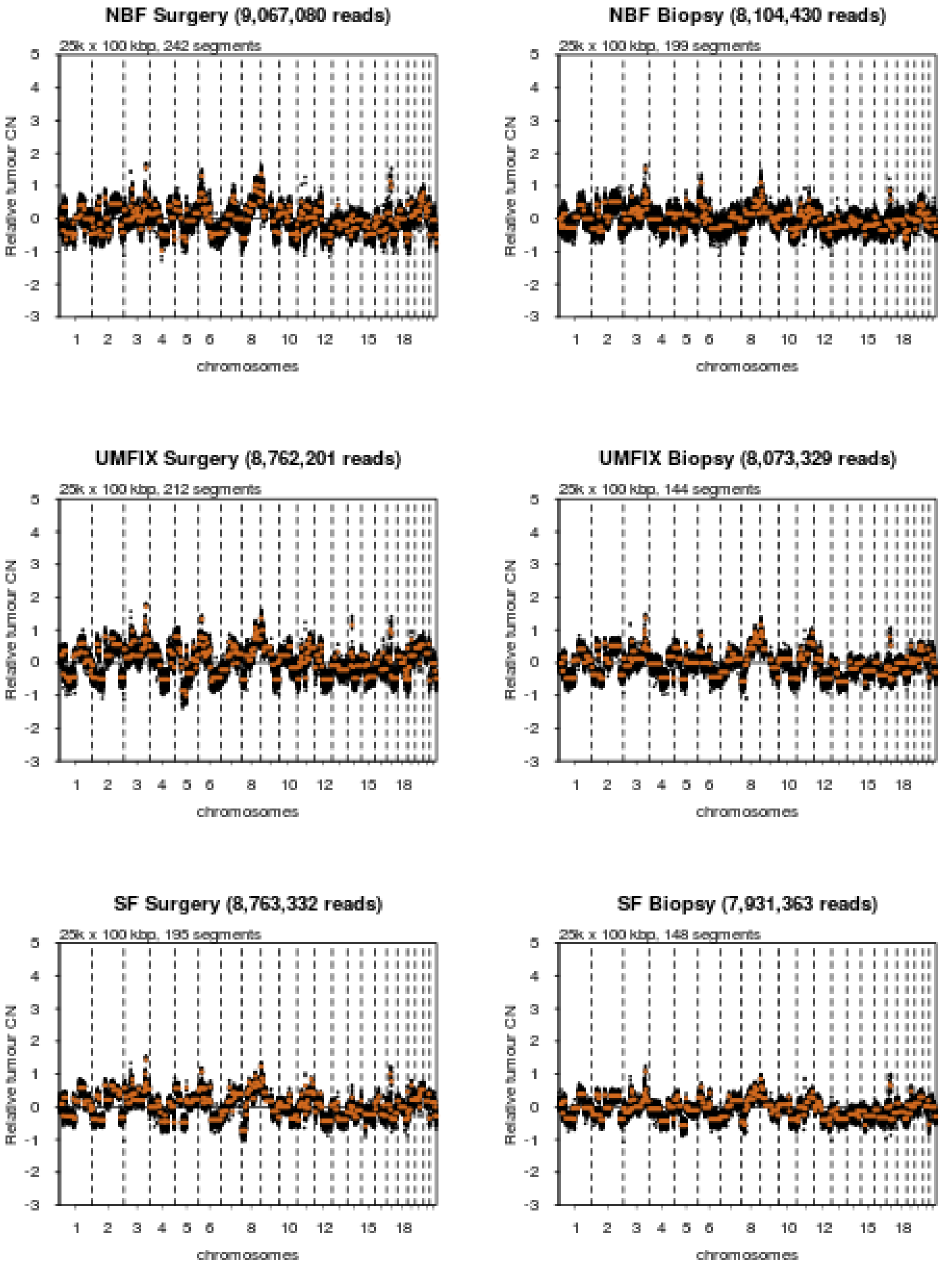


**Supplementary Figure 10:** Copy-number profiles for patient P9

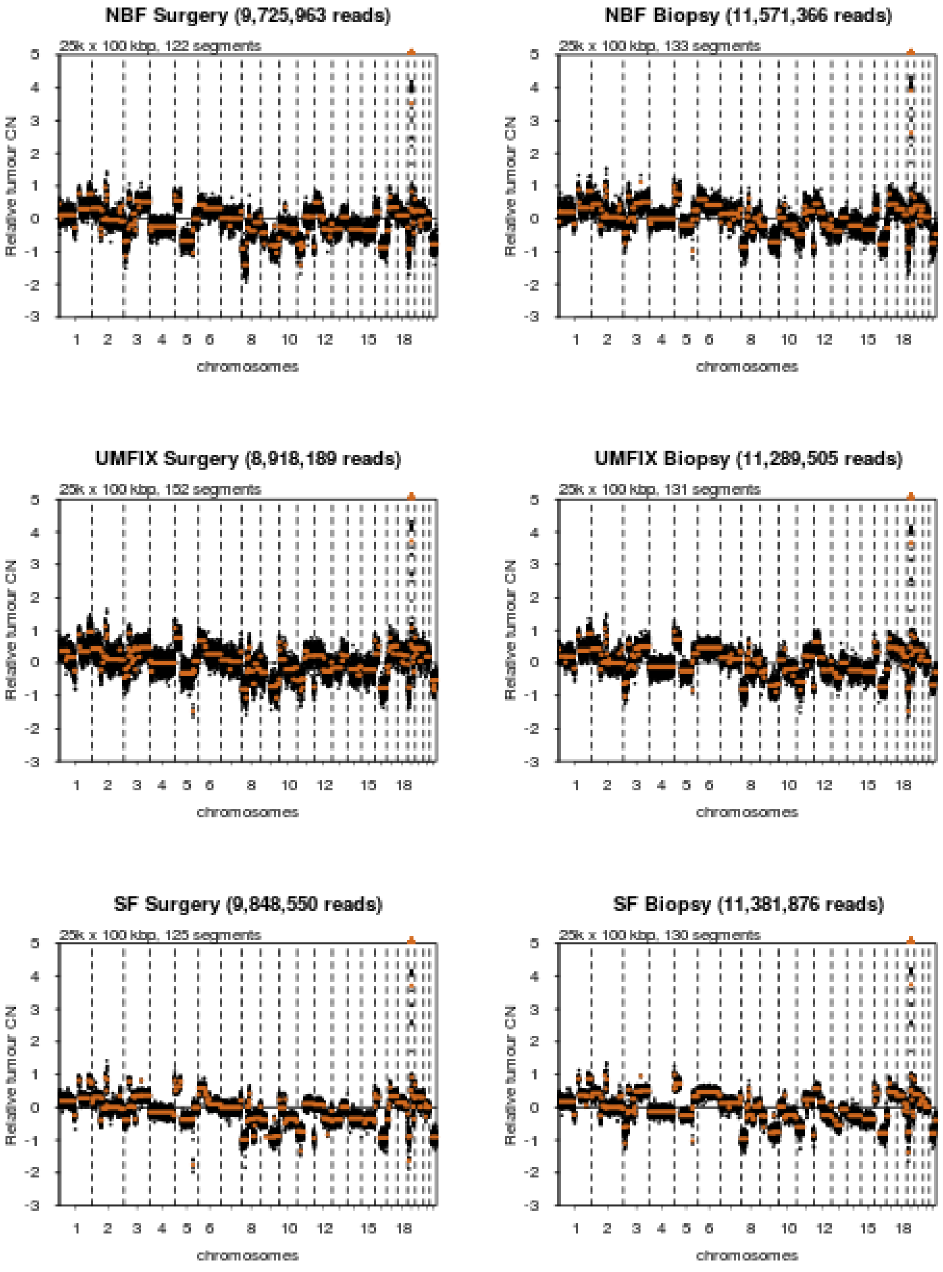


**Supplementary Figure 11:** Copy-number profiles for patient P11

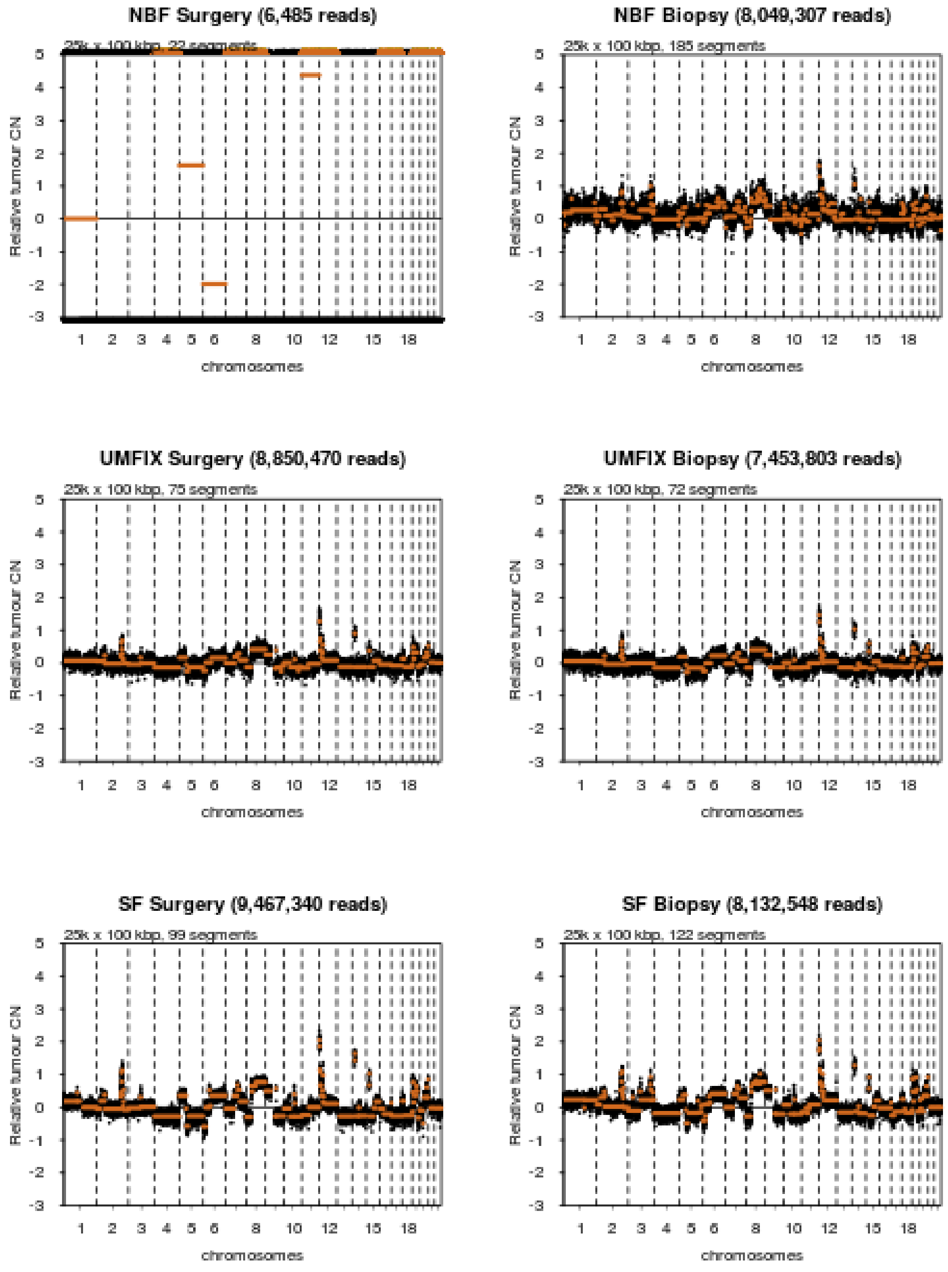


**Supplementary Figure 12:** Copy-number profiles for patient P13

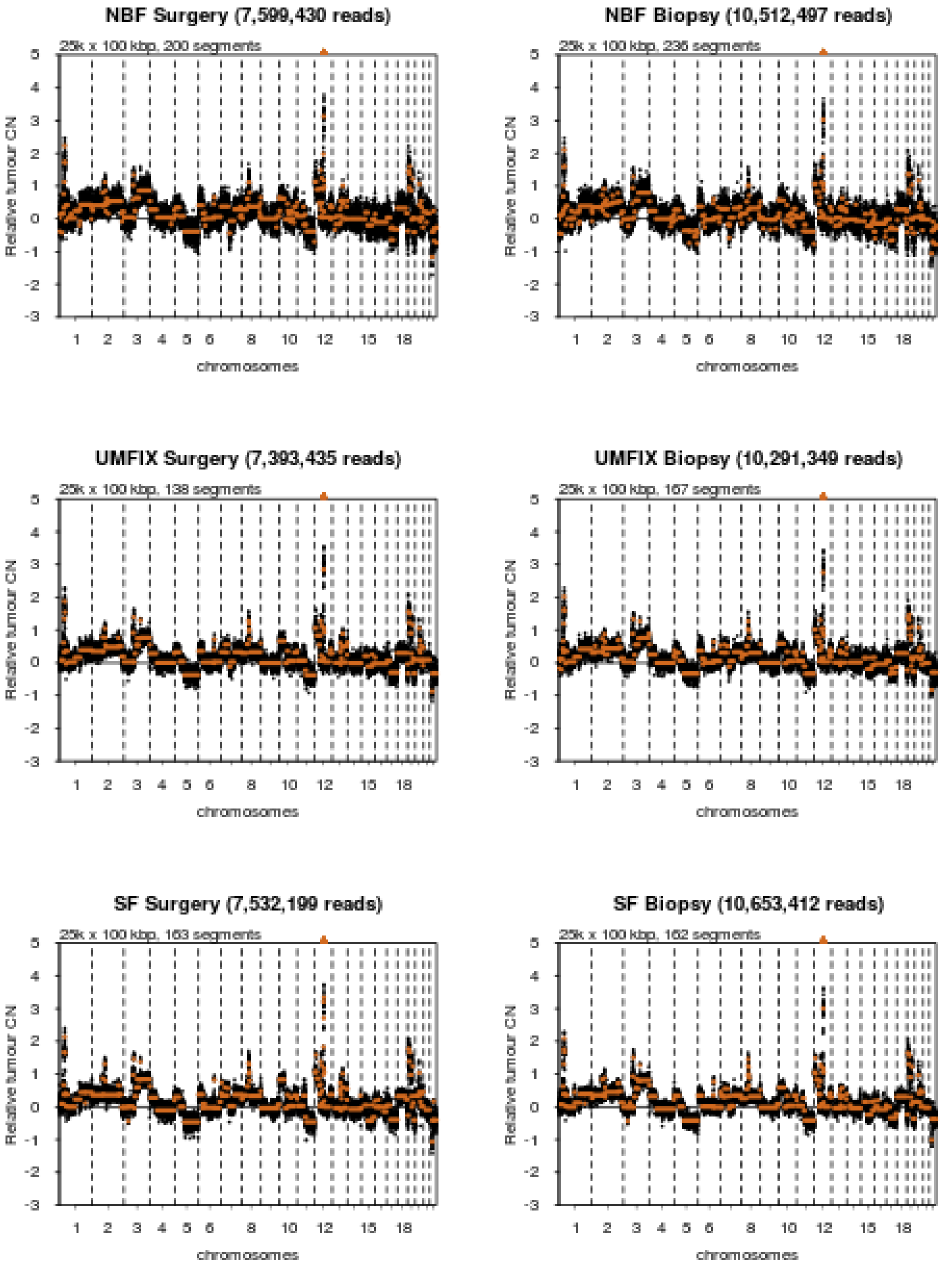


**Supplementary Figure 13:** Copy-number profiles for patient P14

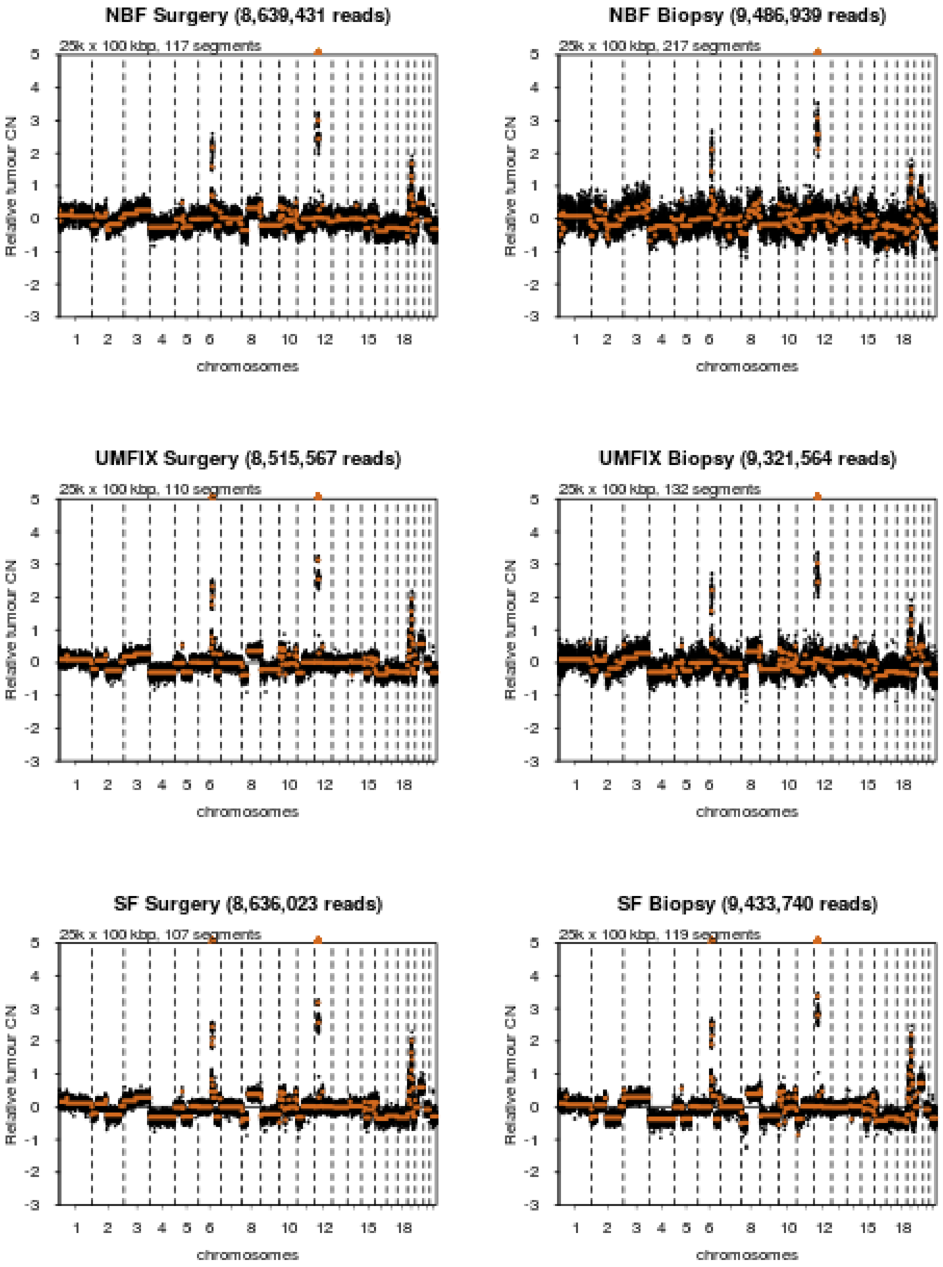


**Supplementary Figure 14:** Copy-number profiles for patient P15

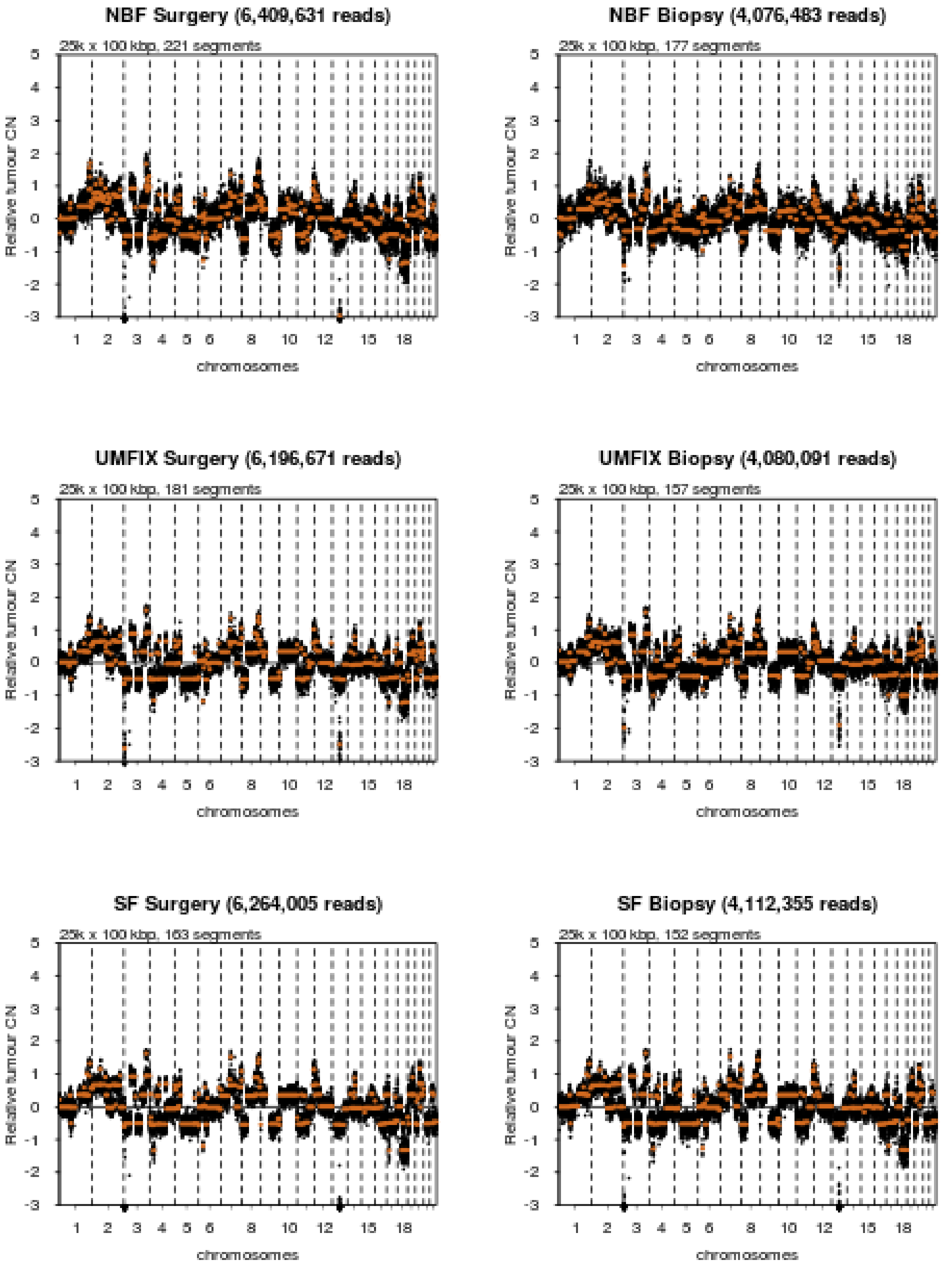


**Supplementary Figure 15:** Copy-number profiles for patient P16

## 6.2 Genome-wide comparison of copy-number calling

Although care was taken to sample tumour material as uniformly as possible for each of the three conditions (UMFIX, NBF and SF), there was naturally some variability in the tumour content of each sample. When generating relative median normalised copy-number profiles, this variation in tumour content manifests as variable deviations from 0 for the same copy-number across conditions. To account for this source of variation, Spearman rank correlation was used as a metric to compare copy-number profiles

as it uses a categorical deviation (e.g. higher or lower) rather than the relative copy-number estimates directly. Another factor confounding comparison was that each sample had a different genome segmentation, making direct comparison of copy-number impossible. Correlation was therefore calculated on a union of all segments identified across conditions to account for differences in the segment numbers. Results are summarised in Supplementary Figure 16.

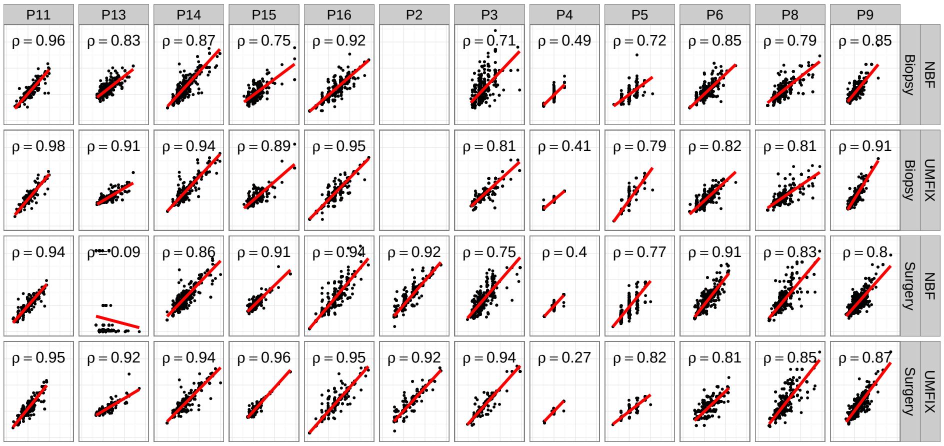


**Supplementary Figure 16:** Comparison of copy-number values (Figure 2c from paper). Scatter plots show correlation between median normalised copy-number profiles from shallow WGS of SF compared to NBF or UMFIX biopsy and surgical samples from 12 patients. Spearman's rank correlation rho is shown.

## 6.3 Comparison of copy-number segment variation.

To look at the accuracy of the copy-number estimate of each segment, we observed the distribution of read depth variance across all segments for samples in UMFIX, NBF or SF. Those with a higher variance are expected to have a higher chance of giving an incorrect copy-number estimate. A summary of the variance observed across 30,104 segments can be found in Supplementary Figure 17.

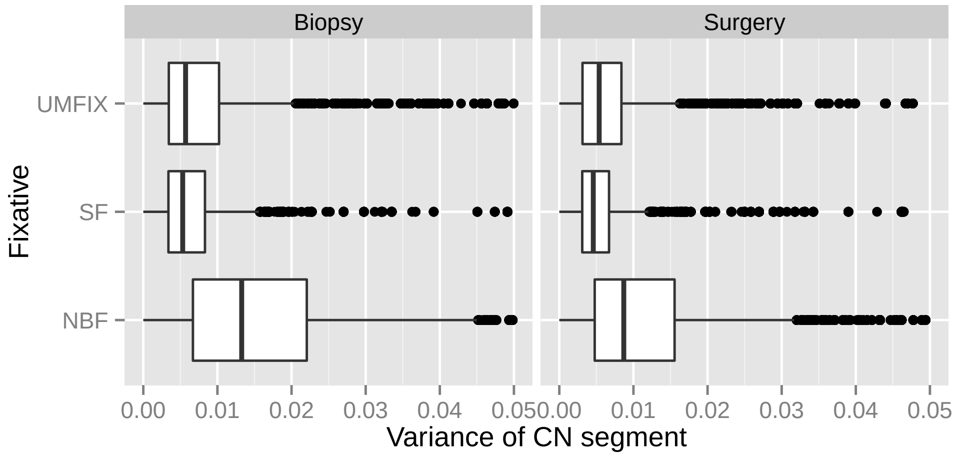


**Supplementary Figure 17:** Comparison of copy-number variance (Figure 2d from paper). Boxplots show observed variance for each copy-number segment (n=90,312) in 69 samples from 12 patients.

*Statistical test:* The Wilcoxon rank-sum test for difference between means was applied to the segment variance for different conditions (plotted in Supplementary Figure 17). The resulting p-values, corrected for multiple testing using the Bonferroni method, are summarised below:

| | UMFIX vs SF | NBF vs SF | NBF vs UMFIX |
|---|---|---|---|
| Biopsy | 4.598574e-71 | 0 | 0 |
| Surgery | 5.192374e-40 | 0 | 0 |

# 7 Tagged-Amplicon Sequencing (TAm-Seq)

Targeted sequencing of 48 amplicons in TP53, PTEN, EGFR, PIK3CA, KRAS and BRAF genes was performed as described previously [7]. All libraries were pooled and quantify using DNA 1000 kit on Agilent 2100 Bioanalyzer and KAPA SYBR® FAST ABI Prism qPCR Kit (KAPA Biosystems) on 7900HT Fast Real-Time PCR System (Applied Biosystems) according to the supplier's recommendations. All samples were analysed in duplicate.

## 7.1 Read alignment and variant calling

Reads were aligned using bwa-mem v0.7.12-r1039 [10] to the 1000 genomes version of human genome build GRCh37, retaining duplicate reads. HaplotypeCaller as part of the Genome Analysis Tool Kit[3] was used to call variants and the Ensembl variant effect predictor[12] used to annotate variants.

## 7.2 Manual curation of SNVs and performance assessment

All reported mutations were verified by visual inspection using the Integrated Genomics Viewer software [18] using the following steps:

- bam files for both technical replicates were uploaded into IGV
- bam files for two independent samples from the same sequencing (but from different patients) run were uploaded into IGV

- manual inspection of each variant was performed by visual comparison of all four bam files

Each SNV was labelled as either a true variant (true positive, TP), or artefact (false positive, FP) using the following criteria

- if the SNV was present in one technical replicate but not the other, the SNV was considered "false positive"
- if the SNV was present at low frequency ($< 10\%$) in the independent samples, the SNV was considered "false positive"
- if the SNV was present at high frequency across all samples from the same sequencing run the SNV was considered "false positive"
- if the SNV was present at very low frequency ($< 1\%$), the SNV was considered "false positive"
- if the SNV was present at high frequency, and not present at a similar frequency in the independent samples (but not across all samples form the same sequencing run) SNV was condsidered "true positive"
- if the SNV was present at low frequency but higher than 5% and present at high frequency or not present in the independent samples the SNV was considered "true positive"

The union of all called SNVs in a patient, across all samples was then used to determine if a SNV was not called but should have been (false negative, FN), or if an artefactual variant was not called (true negative, TN). Two measures were then calulated: sensitivity $TP/(TP + FN)$ and specificity $TN/(TN + FP)$. Figure 18 provides a summary of these metrics for each of the fixatives.

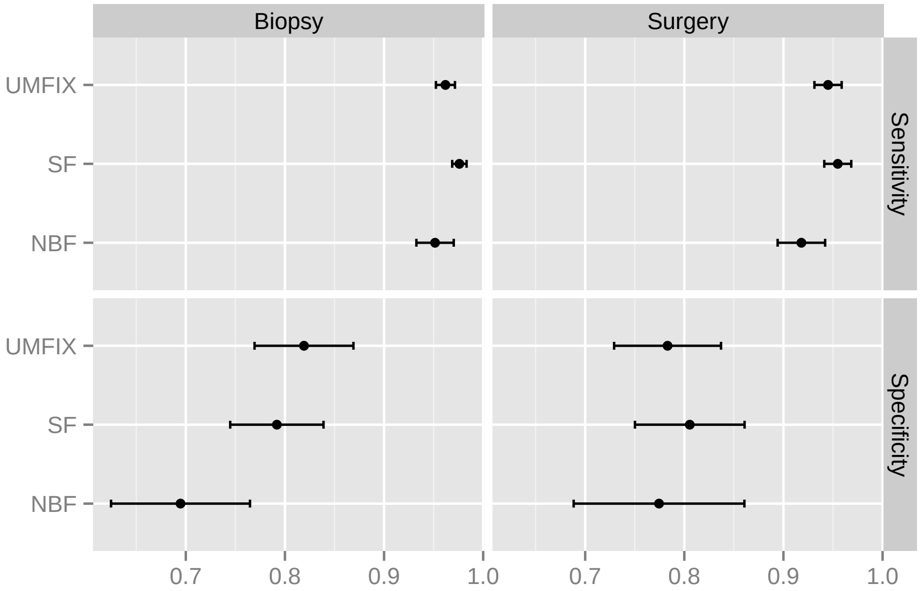


**Supplementary Figure 18:** Observed SNV sensitivity and specificity (Figure 3c from paper). Sensitivity (top) and specificity (bottom) for manually curated SNV calls (n=546) from TAm-SEQ of biopsy and surgical samples from 11 patients. Bars indicate the 95% confidence interval around the indicted mean.

### 7.3 Mutation signature analysis

All non-reference bases appearing in sWGS data were identified using samtools mpileup[11]. Non-reference bases in TAm-Seq data were identified using the h5vc package in Bioconductor[13]. Together these base changes are presumed to be made up of a combination of sequencing artefacts, fixation artefacts, germ-line SNPs, and somatic SNVs. These changes were filtered for putative germline SNPs using dbsnp138, leaving only artefactual changes and true somatic changes (total = 255,376 base changes, see script signature_preprocess.R for details). These base changes were further grouped into four categories: COMMON, those changes found across all conditions (UMFIX, NBF and SF) in a patient; NBF, changes only found in NBF fixed samples; SF, changes only found in SF samples; UMFIX, changes only found in UMFIX samples.

The trinucleotide context of these changes (the bases immediately flanking the change) were extracted from the reference genome and subsequently analysed using the SomaticSignature package in Bioconductor[8]. The SomaticSignature package implements a method, which has been previously applied to identify mutational signatures[1]. These mutational signatures are distinct trinucleotide patterns which may represent a particular mutational force acting on the DNA, such as APOBEC cytosine deamination which occurs in the context of TCW, T and W represent nucleotide abiguity codes[14]. While this technique has been applied to collections of somatic mutations to attempt to understand the underlying mutational mechanisms operational in the tumour, here we use the same idea to model all base changes, predominantly made up of noise, to understand what might be causing this sequencing noise.

Briefly, the observed base changes were summarised by a $C \times S$ frequency matrix which contained the observed frequency of each trinucleotide, for each base change $S$ (96 possible combinations), for each category $C$, (COMMON, NBF, SF, and UMFIX). Non-negative matrix factorisation (NMF) was then applied to this matrix, which decomposed the matrix into two components representing distinct mutational signatures (visually represented in Supplementary Figure 20) and the contribution of each signature to each condition (visually represented in Supplementary Figure 21).

Initally, an assesment had to be made as to how many signatures there were present in the collection of observed base changes. To determine this, NMF was run with 2, 3, and 4 signatures specified and the variance explained by these signatures was observed (Supplementary Figure 19). 3 signatures explained 100% of the variance and as such analysis was run with 3 signatures.

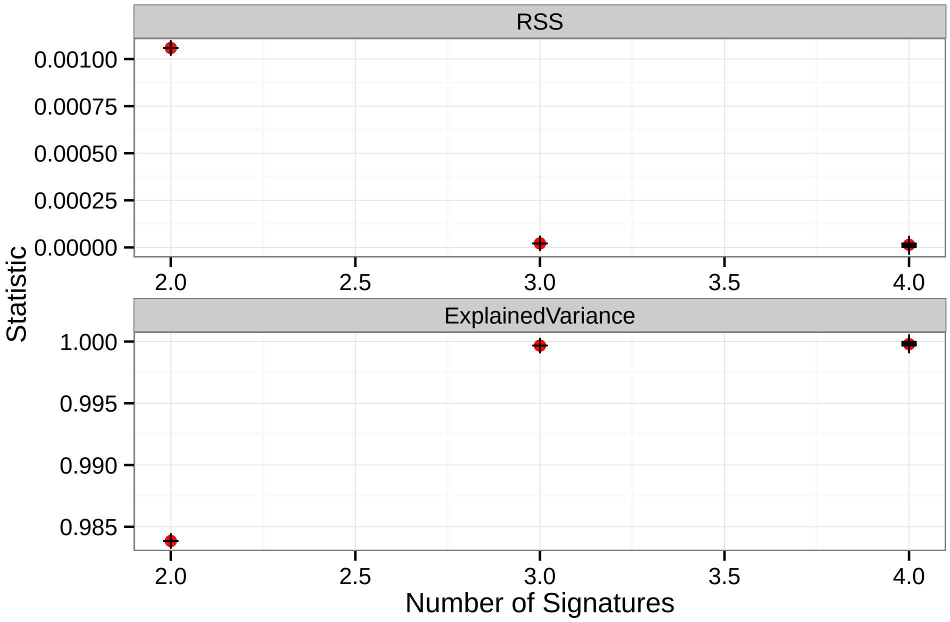


**Supplementary Figure 19:** Number of mutation signatures versus residuals sum of squares (top) and explained variance (bottom).

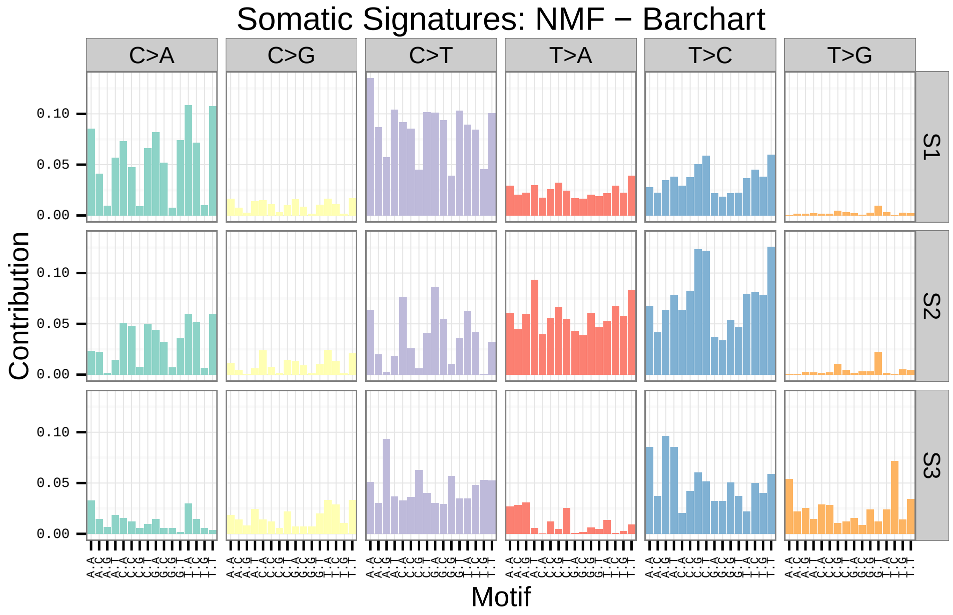


**Supplementary Figure 20:** Mutation signatures (Figure 3a from paper): Bar plots of the 3 somatic mutation signatures (S1-S3) identified by non-negative matrix factorisation using all non-reference bases observed in sWGS and TAm-SEQ sequencing data in 69 samples from 12 patients (n=255,376). Bar plots are grouped by the observed base change with individual bars showing the proportion observed at different tri-nucleotide sequences.

**Supplementary Table 5:** Summary of comparisons between UMFIX and NBF: This table provides an overview of the differences and similarities between UMFIX and NBF along with supporting references.

| Feature | NBF | UMFIX | Evidence |
|---|---|---|---|
| DNA, RNA, protein preservation | poor | good | [9, 2, 6, 17, 20] |
| Changes to DNA, RNA, protein structure | yes | no | [9, 17, 20] |
| DNA amplifiable fragments (>400bp) | no/few | yes/many | [17, 19, 20, 22] |
| DNA molecular weight after fixative exposure (>72hrs) | decreased | unchanged | [19, 20, 22] |
| Artificial DNA mutations | reported | not reported | [21, 16, 4] |
| H&E | good | good | [9, 19, 20] |
| IHC | good | good | [9, 20] |

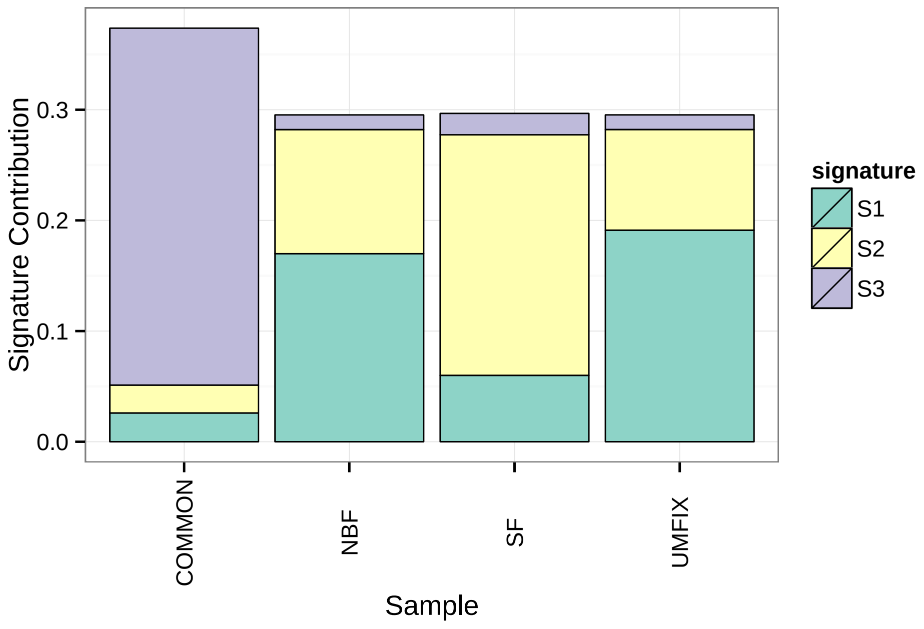


**Supplementary Figure 21:** Contribution of mutation signature (Figure 3b from paper): Stacked bar plots show the proportion of the 3 mutation signatures observed only in SF and NBF or UMFIX fixed samples compared to signatures present in all samples from an individual patient (common)

# 8 Comparison with previous literature

To identify previous studies comparing the performance of UMFIX and NBF for fixation of cancer samples PubMed and Google were used with the following search terms: "fixative and sequencing", "fixative and NGS", "UMFIX", "FFPE and NGS", "UMFIX and sequencing". Key observations made in papers found during this search can be found in Supplementary Table 5. For excellent reviews in the area see [9, 4, 5]

# 9 Instructions for reproducing analysis

In order to compile the supplementary material you will need to undertake the following steps:

- clone the paper repository https:/bitbucket.org/britroc/fixation
- install R (we recommend using RStudio)
- install latex
- install the following R/Bioconductor packages: ggplot2, reshape2, plyr, xlsx, QDNAseq and SomaticSignatures
- compile the sweave document supplementary_material.Rnw

In order to reconstruct the full analysis, bam files and content files should be downloaded from the European Genome Archive, accession EGAS00001001433, and the following scripts modified to point to the directory containing the bam files before running:

- sWGS_downsample.R
- copy_number_calling.R
- base_change_calling.R
- signature_preprocess.R

**Supplementary Table 6:** Histoscores, tumour cellularity and TP53 mutant allele frequency. * - sample stained, but not analysed (missing paired sample), AF = allele frequency

| Patient ID | Fixation | Tissue type | p53 | CK7 | Pax8 | WT1 | CK20 | Cellularity (%) | TP53 mut AF |
|---|---|---|---|---|---|---|---|---|---|
| P1 | NBF | Ctrl | 10 | 2 | 12 | 112 | 0 | <10 | NA |
| P1 | NBF | Tu | 4 | 16 | 14 | 18 | 3 | 70 | NA |
| P1 | UMFIX | Ctrl | 0 | 0 | 48 | 34 | 13 | <10 | NA |
| P1 | UMFIX | Tu | 10 | 128 | 97 | 38 | 7 | 90 | NA |
| P2 | NBF | Ctrl | 0 | 4 | 14 | 141 | 6 | <10 | NA |
| P2 | NBF | Tu | 191 | 98 | 145 | 201 | 0 | 70 | NA |
| P2 | UMFIX | Ctrl | 64 | 4 | 61 | 85 | 0 | <10 | NA |
| P2 | UMFIX | Tu | 168 | 30 | 103 | 99 | 3 | 70 | NA |
| P3 | NBF | Bx | 4 | 105 | 77 | 101 | 5 | 80 | 0.781 |
| P3 | NBF | Ctrl | 2 | 6 | 9 | 16 | 21 | <10 | NA |
| P3 | NBF | Tu | 54 | 124 | 160 | 201 | 3 | 80 | 0.872 |
| P3 | SF | Bx | NA | NA | NA | NA | NA | NA | 0.805 |
| P3 | SF | Tu | NA | NA | NA | NA | NA | NA | 0.779 |
| P3 | UMFIX | Bx | 0 | 108 | 134 | 48 | 1 | 70 | 0.731 |
| P3 | UMFIX | Ctrl | 0 | 12 | 82 | 12 | 15 | <10 | NA |
| P3 | UMFIX | Tu | 8 | 136 | 150 | 166 | 5 | 85 | 0.805 |
| P4 | NBF | Bx | 107 | 98 | 151 | 161 | NA | 40 | 0.697 |
| P4 | NBF | Ctrl | 4 | 0 | 29 | 112 | NA | <10 | NA |
| P4 | NBF | Tu | 132 | 169 | 205 | 200 | NA | 40 | 0.699 |
| P4 | SF | Bx | NA | NA | NA | NA | NA | NA | 0.936 |
| P4 | SF | Tu | NA | NA | NA | NA | NA | NA | 0.812 |
| P4 | UMFIX | Bx | 88 | 104 | 154 | 47 | NA | 30 | 0.539 |
| P4 | UMFIX | Ctrl | 0 | 0 | 73 | 53 | NA | <10 | NA |
| P4 | UMFIX | Tu | 155 | 147 | 167 | 140 | NA | 35 | 0.661 |
| P5 | NBF | Bx | 148 | 10 | 130 | 5 | 3 | 95 | 0.194 |
| P5 | NBF | Ctrl | 6 | 13 | 39 | 37 | 12 | <10 | NA |
| P5 | NBF | Tu | 179 | 22 | 142 | 168 | 0 | 70 | 0.604 |
| P5 | SF | Bx | NA | NA | NA | NA | NA | NA | 0.528 |
| P5 | SF | Tu | NA | NA | NA | NA | NA | NA | 0.606 |
| P5 | UMFIX | Bx | 163 | 6 | 104 | 100 | 3 | 90 | 0.631 |
| P5 | UMFIX | Ctrl | 2 | 16 | 41 | 12 | 1 | <10 | NA |
| P5 | UMFIX | Tu | 84 | 0 | 67 | 68 | 0 | 70 | 0.261 |
| P6 | NBF | Bx | 152 | 63 | NA | 47 | 3 | 80 | 0.535 |
| P6 | NBF | Ctrl | 0 | 0 | 7 | 65 | 0 | <10 | NA |
| P6 | NBF | Tu | 168 | 100 | 93 | 203 | 1 | 95 | 0.624 |
| P6 | SF | Bx | NA | NA | NA | NA | NA | NA | 0.631 |
| P6 | SF | Tu | NA | NA | NA | NA | NA | NA | 0.620 |
| P6 | UMFIX | Bx | 151 | 50 | 161* | 108 | 12 | 95 | 0.604 |
| P6 | UMFIX | Ctrl | 0 | 0 | 54 | 96 | 0 | <10 | NA |
| P6 | UMFIX | Tu | 132 | 76 | 103 | 176 | 0 | 95 | 0.489 |
| P7 | NBF | Ctrl | 2 | 0 | 11 | 12 | 20 | <10 | NA |
| P7 | NBF | Tu | 60 | 124 | 153 | 175 | 36 | 85 | NA |
| P7 | UMFIX | Ctrl | 0 | 0 | 25 | 11 | 38 | <10 | NA |
| P7 | UMFIX | Tu | 32 | 110 | 123 | 103 | 3 | 80 | NA |
| P8 | NBF | Bx | 117* | 73 | 107 | 109 | NA | 70 | 0.737 |
| P8 | NBF | Ctrl | 6 | 0 | 10 | 100 | NA | <10 | NA |
| P8 | NBF | Tu | 183 | 142 | 156 | 41 | NA | 70 | 0.850 |
| P8 | SF | Bx | NA | NA | NA | NA | NA | NA | 0.853 |

| | | | | | | | | | |
|---|---|---|---|---|---|---|---|---|---|
| P8 | SF | Tu | NA | NA | NA | NA | NA | NA | 0.773 |
| P8 | UMFIX | Bx | NA | 59 | 146 | 32 | NA | 65 | 0.675 |
| P8 | UMFIX | Ctrl | 2 | 4 | 105 | 72 | NA | <10 | NA |
| P8 | UMFIX | Tu | 195 | 145 | 166 | 69 | NA | 90 | 0.863 |
| P9 | NBF | Bx | 131 | 82 | 100 | 45 | 12 | NA | NA |
| P9 | NBF | Bx | 46 | 10 | 32 | 49 | 0 | 45 | 0.446 |
| P9 | NBF | Ctrl | 2 | 2 | 18 | 8 | 3 | <10 | NA |
| P9 | NBF | Tu | 170 | 105 | 165 | 0 | 2 | 95 | 0.777 |
| P9 | SF | Bx | NA | NA | NA | NA | NA | NA | 0.551 |
| P9 | SF | Tu | NA | NA | NA | NA | NA | NA | 0.682 |
| P9 | UMFIX | Bx | 61 | 26 | 81 | 28 | 0 | NA | NA |
| P9 | UMFIX | Bx | 56 | 31 | 113 | 65 | 0 | 60 | 0.653 |
| P9 | UMFIX | Ctrl | 4 | 2 | 35 | 0 | 6 | <10 | NA |
| P9 | UMFIX | Tu | 168 | 84 | 117 | 168 | 35 | 90 | 0.821 |
| P10 | NBF | Ctrl | 6 | 0 | 19 | 95 | 0 | <10 | NA |
| P10 | NBF | Tu | 94 | 75 | 106 | 76 | 7 | 60 | NA |
| P10 | UMFIX | Ctrl | 68 | 6 | 52 | 72 | 3 | <10 | NA |
| P10 | UMFIX | Tu | 19 | 12 | 86 | 24 | 7 | 50 | NA |
| P11 | NBF | Bx | 183 | 128 | 55 | 75 | 6 | 70 | 0.848 |
| P11 | NBF | Ctrl | 16 | 32 | 32 | 70 | 117 | <10 | NA |
| P11 | NBF | Tu | 183 | 128 | 88 | 185 | 3 | 65 | 0.880 |
| P11 | SF | Bx | NA | NA | NA | NA | NA | NA | 0.922 |
| P11 | SF | Tu | NA | NA | NA | NA | NA | NA | 0.930 |
| P11 | UMFIX | Bx | 156 | 138 | 88 | 40 | 5 | 70 | 0.846 |
| P11 | UMFIX | Ctrl | 10 | 17 | 62 | 17 | 31 | <10 | NA |
| P11 | UMFIX | Tu | 161 | 106 | 93 | 60 | 2 | 70 | 0.867 |
| P12 | NBF | Bx | NA | NA | NA | NA | NA | 30 | 0.413 |
| P12 | NBF | Tu | NA | NA | NA | NA | NA | 30 | 0.333 |
| P12 | SF | Bx | NA | NA | NA | NA | NA | NA | 0.439 |
| P12 | SF | Tu | NA | NA | NA | NA | NA | NA | 0.659 |
| P12 | UMFIX | Bx | NA | NA | NA | NA | NA | 30 | 0.480 |
| P12 | UMFIX | Tu | NA | NA | NA | NA | NA | 27 | 0.360 |
| P13 | NBF | Bx | 22 | 25 | 169 | 56 | 8 | NA | 0.236 |
| P13 | NBF | Ctrl | 8 | 25 | 65 | 109 | 2 | <10 | NA |
| P13 | NBF | Tu | 16 | 37 | 146 | 59 | 9 | NA | 0.541 |
| P13 | SF | Bx | NA | NA | NA | NA | NA | NA | 0.393 |
| P13 | SF | Tu | NA | NA | NA | NA | NA | NA | 0.321 |
| P13 | UMFIX | Bx | 0 | 74 | 122 | 3 | 1 | NA | 0.224 |
| P13 | UMFIX | Ctrl | 28 | 20 | 97 | 57 | 3 | <10 | NA |
| P13 | UMFIX | Tu | 16 | 20 | 112 | 28 | 16 | NA | 0.192 |
| P14 | NBF | Bx | 110 | 105 | 172 | 112 | 19 | 70 | NA |
| P14 | NBF | Ctrl | 16 | 2* | 31 | 10 | 23 | <10 | NA |
| P14 | NBF | Tu | 150 | 149 | 192 | 132 | 9 | 80 | NA |
| P14 | UMFIX | Bx | 92 | 80 | 170 | 21 | 3 | 70 | NA |
| P14 | UMFIX | Ctrl | 6 | NA | 81 | 0 | 5 | <10 | NA |
| P14 | UMFIX | Tu | 108 | 120 | 153 | 115 | 5 | 60 | NA |
| P15 | NBF | Bx | 2 | 47 | 94 | 5 | 4 | 30 | 0.406 |
| P15 | NBF | Ctrl | 0 | 4 | 24 | 50 | 5 | <10 | NA |
| P15 | NBF | Tu | 8 | 68 | 80 | 13 | 1 | 40 | 0.377 |
| P15 | SF | Bx | NA | NA | NA | NA | NA | NA | 0.622 |
| P15 | SF | Tu | NA | NA | NA | NA | NA | NA | 0.536 |
| P15 | UMFIX | Bx | 0 | 125 | 119 | 0 | 18 | 50 | 0.412 |
| P15 | UMFIX | Ctrl | 0 | 19 | 67 | 17 | 3 | <10 | NA |
| P15 | UMFIX | Tu | 2 | 76 | 87 | 3 | 38 | 50 | 0.509 |
| P16 | NBF | Bx | 157 | 119 | 139 | 167 | 5 | NA | 0.715 |
| P16 | NBF | Ctrl | 0 | 4 | 16 | 84* | 4 | <10 | NA |
| P16 | NBF | Tu | 186 | 147* | 178 | 187 | 0 | 90 | 0.908 |
| P16 | SF | Bx | NA | NA | NA | NA | NA | NA | 0.925 |
| P16 | SF | Tu | NA | NA | NA | NA | NA | NA | 0.908 |
| P16 | UMFIX | Bx | 64 | 26 | 103 | 34 | 15 | NA | 0.794 |
| P16 | UMFIX | Ctrl | 0 | 2 | 62 | NA | 3 | <10 | NA |
| P16 | UMFIX | Tu | 148 | NA | 119 | 108 | 3 | 85 | 0.881 |

## **Supplementary Table 7:** REMARK table

| Patient ID | Fixation | Tissue | DNA QC | sWGS | TAm-Seq | H&E | IHC-p53 | IHC-CK7 | IHC-Pax8 | IHC-WT1 | IHC-CK20 |
|---|---|---|---|---|---|---|---|---|---|---|---|
| P1 | NBF | Ctrl | N | N | N | Y | Y | Y | Y | Y | Y |
| P1 | NBF | Tu | N | N | N | Y | Y | Y | Y | Y | Y |
| P1 | UMFIX | Ctrl | N | N | N | Y | Y | Y | Y | Y | Y |
| P1 | UMFIX | Tu | N | N | N | Y | Y | Y | Y | Y | Y |
| P2 | NBF | Ctrl | N | N | N | Y | Y | Y | Y | Y | Y |
| P2 | NBF | Tu | N | Y | N | Y | Y | Y | Y | Y | Y |
| P2 | SF | Tu | N | Y | N | N | N | N | N | N | N |
| P2 | UMFIX | Ctrl | N | N | N | Y | Y | Y | Y | Y | Y |
| P2 | UMFIX | Tu | N | Y | N | Y | Y | Y | Y | Y | Y |
| P3 | NBF | Bx | Y | Y | Y | Y | Y | Y | Y | Y | Y |
| P3 | NBF | Ctrl | N | N | N | Y | Y | Y | Y | Y | Y |
| P3 | NBF | Tu | Y | Y | Y | Y | Y | Y | Y | Y | Y |
| P3 | SF | Bx | Y | Y | Y | N | N | N | N | N | N |
| P3 | SF | Tu | Y | Y | Y | N | N | N | N | N | N |
| P3 | UMFIX | Bx | Y | Y | Y | Y | Y | Y | Y | Y | Y |
| P3 | UMFIX | Ctrl | N | N | N | Y | Y | Y | Y | Y | Y |
| P3 | UMFIX | Tu | Y | Y | Y | Y | Y | Y | Y | Y | Y |
| P4 | NBF | Bx | Y | Y | Y | Y | Y | Y | Y | Y | N |
| P4 | NBF | Ctrl | N | N | N | Y | Y | Y | Y | Y | N |
| P4 | NBF | Tu | Y | Y | Y | Y | Y | Y | Y | Y | N |
| P4 | SF | Bx | Y | Y | Y | N | N | N | N | N | N |

| | | | | | | | | | | | |
|---|---|---|---|---|---|---|---|---|---|---|---|
| P4 | SF | Tu | Y | Y | Y | N | N | N | N | N | N |
| P4 | UMFIX | Bx | Y | Y | Y | Y | Y | Y | Y | Y | N |
| P4 | UMFIX | Ctrl | N | N | N | Y | Y | Y | Y | Y | N |
| P4 | UMFIX | Tu | Y | Y | Y | Y | Y | Y | Y | Y | N |
| P5 | NBF | Bx | Y | Y | Y | Y | Y | Y | Y | Y | Y |
| P5 | NBF | Ctrl | N | N | N | Y | Y | Y | Y | Y | Y |
| P5 | NBF | Tu | Y | Y | Y | Y | Y | Y | Y | Y | Y |
| P5 | SF | Bx | Y | Y | Y | N | N | N | N | N | N |
| P5 | SF | Tu | Y | Y | Y | N | N | N | N | N | N |
| P5 | UMFIX | Bx | Y | Y | Y | Y | Y | Y | Y | Y | Y |
| P5 | UMFIX | Ctrl | N | N | N | Y | Y | Y | Y | Y | Y |
| P5 | UMFIX | Tu | Y | Y | Y | Y | Y | Y | Y | Y | Y |
| P6 | NBF | Bx | Y | Y | Y | Y | Y | Y | N | Y | Y |
| P6 | NBF | Ctrl | N | N | N | Y | Y | Y | Y | Y | Y |
| P6 | NBF | Tu | Y | Y | Y | Y | Y | Y | Y | Y | Y |
| P6 | SF | Bx | Y | Y | Y | N | N | N | N | N | N |
| P6 | SF | Tu | Y | Y | Y | N | N | N | N | N | N |
| P6 | UMFIX | Bx | Y | Y | Y | Y | Y | Y | N | Y | Y |
| P6 | UMFIX | Ctrl | N | N | N | Y | Y | Y | Y | Y | Y |
| P6 | UMFIX | Tu | Y | Y | Y | Y | Y | Y | Y | Y | Y |
| P7 | NBF | Ctrl | N | N | N | Y | Y | Y | Y | Y | Y |
| P7 | NBF | Tu | N | N | N | Y | Y | Y | Y | Y | Y |
| P7 | UMFIX | Ctrl | N | N | N | Y | Y | Y | Y | Y | Y |
| P7 | UMFIX | Tu | N | N | N | Y | Y | Y | Y | Y | Y |
| P8 | NBF | Bx | Y | Y | Y | Y | N | Y | Y | Y | N |
| P8 | NBF | Ctrl | N | N | N | Y | Y | Y | Y | Y | N |
| P8 | NBF | Tu | Y | Y | Y | Y | Y | Y | Y | Y | N |
| P8 | SF | Bx | Y | Y | Y | N | N | N | N | N | N |
| P8 | SF | Tu | Y | Y | Y | N | N | N | N | N | N |
| P8 | UMFIX | Bx | Y | Y | Y | Y | N | Y | Y | Y | N |
| P8 | UMFIX | Ctrl | N | N | N | Y | Y | Y | Y | Y | N |
| P8 | UMFIX | Tu | Y | Y | Y | Y | Y | Y | Y | Y | N |
| P9 | NBF | Bx | N | N | N | Y | Y | Y | Y | Y | Y |
| P9 | NBF | Bx | Y | Y | Y | Y | Y | Y | Y | Y | Y |
| P9 | NBF | Bx | N | N | N | Y | N | N | N | N | N |
| P9 | NBF | Ctrl | N | N | N | Y | Y | Y | Y | Y | Y |
| P9 | NBF | Tu | Y | Y | Y | Y | Y | Y | Y | Y | Y |
| P9 | SF | Bx | Y | Y | Y | N | N | N | N | N | N |
| P9 | SF | Tu | Y | Y | Y | N | N | N | N | N | N |
| P9 | UMFIX | Bx | N | N | N | Y | Y | Y | Y | Y | Y |
| P9 | UMFIX | Bx | Y | Y | Y | Y | Y | Y | Y | Y | Y |
| P9 | UMFIX | Ctrl | N | N | N | Y | Y | Y | Y | Y | Y |
| P9 | UMFIX | Tu | Y | Y | Y | Y | Y | Y | Y | Y | Y |
| P10 | NBF | Ctrl | N | N | N | Y | Y | Y | Y | Y | Y |
| P10 | NBF | Tu | N | N | N | Y | Y | Y | Y | Y | Y |
| P10 | UMFIX | Ctrl | N | N | N | Y | Y | Y | Y | Y | Y |
| P10 | UMFIX | Tu | N | N | N | Y | Y | Y | Y | Y | Y |
| P11 | NBF | Bx | Y | Y | Y | Y | Y | Y | Y | Y | Y |
| P11 | NBF | Ctrl | N | N | N | Y | Y | Y | Y | Y | Y |
| P11 | NBF | Tu | Y | Y | Y | Y | Y | Y | Y | Y | Y |
| P11 | SF | Bx | Y | Y | Y | N | N | N | N | N | N |
| P11 | SF | Tu | Y | Y | Y | N | N | N | N | N | N |
| P11 | UMFIX | Bx | Y | Y | Y | Y | Y | Y | Y | Y | Y |
| P11 | UMFIX | Ctrl | N | N | N | Y | Y | Y | Y | Y | Y |
| P11 | UMFIX | Tu | Y | Y | Y | Y | Y | Y | Y | Y | Y |
| P12 | NBF | Bx | N | N | Y | N | N | N | N | N | N |
| P12 | NBF | Tu | N | N | Y | N | N | N | N | N | N |
| P12 | SF | Bx | N | N | Y | N | N | N | N | N | N |
| P12 | SF | Tu | N | N | Y | N | N | N | N | N | N |
| P12 | UMFIX | Bx | N | N | Y | N | N | N | N | N | N |
| P12 | UMFIX | Tu | N | N | Y | N | N | N | N | N | N |
| P13 | NBF | Bx | Y | Y | Y | Y | Y | Y | Y | Y | Y |
| P13 | NBF | Ctrl | N | N | N | Y | Y | Y | Y | Y | Y |
| P13 | NBF | Tu | Y | Y | Y | Y | Y | Y | Y | Y | Y |
| P13 | SF | Bx | Y | Y | Y | N | N | N | N | N | N |
| P13 | SF | Tu | Y | Y | Y | N | N | N | N | N | N |
| P13 | UMFIX | Bx | Y | Y | Y | Y | Y | Y | Y | Y | Y |
| P13 | UMFIX | Ctrl | N | N | N | Y | Y | Y | Y | Y | Y |
| P13 | UMFIX | Tu | Y | Y | Y | Y | Y | Y | Y | Y | Y |
| P14 | NBF | Bx | Y | Y | N | Y | Y | Y | Y | Y | Y |
| P14 | NBF | Ctrl | N | N | N | Y | Y | N | Y | Y | Y |
| P14 | NBF | Tu | Y | Y | N | Y | Y | Y | Y | Y | Y |
| P14 | NBF | Tu | N | N | N | Y | N | N | N | N | N |
| P14 | SF | Bx | Y | Y | N | N | N | N | N | N | N |
| P14 | SF | Tu | Y | Y | N | N | N | N | N | N | N |
| P14 | UMFIX | Bx | Y | Y | N | Y | Y | Y | Y | Y | Y |
| P14 | UMFIX | Ctrl | N | N | N | Y | Y | N | Y | Y | Y |
| P14 | UMFIX | Tu | Y | Y | N | Y | Y | Y | Y | Y | Y |
| P15 | NBF | Bx | Y | Y | Y | Y | Y | Y | Y | Y | Y |
| P15 | NBF | Ctrl | N | N | N | Y | Y | Y | Y | Y | Y |
| P15 | NBF | Tu | Y | Y | Y | Y | Y | Y | Y | Y | Y |
| P15 | SF | Bx | Y | Y | Y | N | N | N | N | N | N |
| P15 | SF | Tu | Y | Y | Y | N | N | N | N | N | N |
| P15 | UMFIX | Bx | Y | Y | Y | Y | Y | Y | Y | Y | Y |
| P15 | UMFIX | Ctrl | N | N | N | Y | Y | Y | Y | Y | Y |
| P15 | UMFIX | Tu | Y | Y | Y | Y | Y | Y | Y | Y | Y |
| P15 | UMFIX | Tu | N | N | N | Y | N | N | N | N | N |
| P16 | NBF | Bx | Y | Y | Y | Y | Y | Y | Y | Y | Y |
| P16 | NBF | Ctrl | N | N | N | Y | Y | Y | Y | N | Y |
| P16 | NBF | Tu | Y | Y | Y | Y | Y | N | Y | Y | Y |
| P16 | SF | Bx | Y | Y | Y | N | N | N | N | N | N |
| P16 | SF | Tu | Y | Y | Y | N | N | N | N | N | N |

| | | | | | | | | | | | |
|---|---|---|---|---|---|---|---|---|---|---|---|
| P16 | UMFIX | Bx | Y | Y | Y | Y | Y | Y | Y | Y | Y |
| P16 | UMFIX | Ctrl | N | N | N | Y | Y | Y | Y | N | Y |
| P16 | UMFIX | Tu | Y | Y | Y | Y | Y | N | Y | Y | Y |

# References


[1] Ludmil B Alexandrov et al. "Signatures of mutational processes in human cancer." en. In: *Nature* 500.7463 (Aug. 2013), pp. 415–21. ISSN: 1476-4687. DOI: 10.1038/nature12477. URL: http://www.nature.com/nature/journal/vaop/ncurrent/full/nature12477.html?goback=.gde_2130872_member_266007157.

[2] Melissa L. Cox et al. "Assessment of fixatives, fixation, and tissue processing on morphology and RNA integrity". In: *Experimental and Molecular Pathology* 80 (2006), pp. 183–191. ISSN: 00144800. DOI: 10.1016/j.yexmp.2005.10.002.

[3] Mark A DePristo et al. "A framework for variation discovery and genotyping using next-generation DNA sequencing data." In: *Nature genetics* 43.5 (May 2011), pp. 491–8. ISSN: 1546-1718. URL: http://dx.doi.org/10.1038/ng.806.

[4] H. Do and A. Dobrovic. "Sequence Artifacts in DNA from Formalin-Fixed Tissues: Causes and Strategies for Minimization". In: *Clinical Chemistry* 61.1 (2014), pp. 64–71. ISSN: 0009-9147. DOI: 10.1373/clinchem.2014.223040. URL: http://www.clinchem.org/cgi/doi/10.1373/clinchem.2014.223040.

[5] Hongdo Do et al. "Reducing sequence artifacts in amplicon-based massively parallel sequencing of formalin-fixed paraffin-embedded DNA by enzymatic depletion of uracil-containing templates". In: *Clinical Chemistry* 59.9 (2013), pp. 1376–1383. ISSN: 00099147. DOI: 10.1373/clinchem.2012.202390.

[6] Michael P. Douglas and Scott O. Rogers. "DNA damage caused by common cytological fixatives". In: *Mutation Research - Fundamental and Molecular Mechanisms of Mutagenesis* 401.1-2 (1998), pp. 77–88. ISSN: 00275107. DOI: 10.1016/S0027-5107(97)00314-X.

[7] Tim Forshew et al. "Noninvasive identification and monitoring of cancer mutations by targeted deep sequencing of plasma DNA." In: *Science translational medicine* 4.136 (May 2012), 136ra68. ISSN: 1946-6242. DOI: 10.1126/scitranslmed.3003726. URL: http://www.ncbi.nlm.nih.gov/pubmed/22649089.

[8] Julian S. Gehring et al. "SomaticSignatures: Inferring Mutational Signatures from Single Nucleotide Variants". In: *Bioinformatics* (July 2015), btv408. ISSN: 1367-4803. URL: http://bioinformatics.oxfordjournals.org/content/early/2015/08/13/bioinformatics.btv408.

[9] William J Howat and Beverley a Wilson. "Tissue fixation and the effect of molecular fixatives on downstream staining procedures." In: *Methods (San Diego, Calif.)* 70.1 (2014), pp. 12–19. ISSN: 1095-9130. DOI: 10.1016/j.ymeth.2014.01.022. URL: http://www.ncbi.nlm.nih.gov/pubmed/24561827.

[10] Heng Li. "Aligning sequence reads, clone sequences and assembly contigs with BWA-MEM". In: (Mar. 2013), p. 3. URL: http://arxiv.org/abs/1303.3997.

[11] Heng Li et al. "The Sequence Alignment/Map format and SAMtools." In: *Bioinformatics (Oxford, England)* 25.16 (Aug. 2009), pp. 2078–9. ISSN: 1367-4811. URL: http://www.ncbi.nlm.nih.gov/pubmed/19505943.

[12] William McLaren et al. "Deriving the consequences of genomic variants with the Ensembl API and SNP Effect Predictor." In: *Bioinformatics (Oxford, England)* 26.16 (Aug. 2010), pp. 2069–70. ISSN: 1367-4811. URL: http://www.pubmedcentral.nih.gov/articlerender.fcgi?artid=2916720\&tool=pmcentrez\&rendertype=abstract.

[13] Paul Theodor Pyl et al. "h5vc: scalable nucleotide tallies with HDF5." In: *Bioinformatics (Oxford, England)* 30.10 (May 2014), pp. 1464–6. ISSN: 1367-4811. URL: http://bioinformatics.oxfordjournals.org/content/30/10/1464.

[14] Steven A Roberts et al. "An APOBEC cytidine deaminase mutagenesis pattern is widespread in human cancers." en. In: *Nature genetics* 45.9 (Sept. 2013), pp. 970–6. ISSN: 1546-1718. URL: http://www.nature.com/ng/journal/v45/n9/full/ng.2702.html?WT.ec_id=NG-201309.

[15] Ilari Scheinin et al. "DNA copy number analysis of fresh and formalin-fixed specimens by shallow whole-genome sequencing with identification and exclusion of problematic regions in the genome assembly". In: *Genome Research* 24.12 (Dec. 2014), pp. 2022–2032. ISSN: 1088-9051. DOI: 10.1101/gr.175141.114. URL: http://genome.cshlp.org/content/24/12/2022.short.

[16] David H. Spencer et al. "Comparison of clinical targeted next-generation sequence data from formalin-fixed and fresh-frozen tissue specimens". In: *Journal of Molecular Diagnostics* 15.5 (2013), pp. 623–633. ISSN: 15251578. DOI: 10.1016/j.jmoldx.2013.05.004. URL: http://dx.doi.org/10.1016/j.jmoldx.2013.05.004.

[17] Mythily Srinivasan, Daniel Sedmak, and Scott Jewell. "Effect of fixatives and tissue processing on the content and integrity of nucleic acids." In: *The American journal of pathology* 161.6 (2002), pp. 1961–1971. ISSN: 00029440. DOI: 10.1016/S0002-9440(10)64472-0. URL: http://dx.doi.org/10.1016/S0002-9440(10)64472-0.

[18] Helga Thorvaldsdóttir, James T Robinson, and Jill P Mesirov. "Integrative Genomics Viewer (IGV): high-performance genomics data visualization and exploration." In: *Briefings in bioinformatics* 14.2 (Mar. 2013), pp. 178–92. ISSN: 1477-4054. URL: http://bib.oxfordjournals.org/content/14/2/178.full?keytype=ref\&ijkey=qTgjFwbRBAzRZWC.

[19] Gulisa Turashvili et al. "Nucleic acid quantity and quality from paraffin blocks: Defining optimal fixation, processing and DNA/RNA extraction techniques". In: *Experimental and Molecular Pathology* 92.1 (2012), pp. 33–43. ISSN: 00144800. DOI: 10.1016/j.yexmp.2011.09.013. URL: http://dx.doi.org/10.1016/j.yexmp.2011.09.013.

[20] Vladimir Vincek et al. "A tissue fixative that protects macromolecules (DNA, RNA, and protein) and histomorphology in clinical samples." In: *Laboratory investigation; a journal of technical methods and pathology* 83.10 (2003), pp. 1427–1435. ISSN: 0023-6837. DOI: 10.1097/01.LAB.0000090154.55436.D1.

[21] C Williams et al. "A high frequency of sequence alterations is due to formalin fixation of archival specimens." In: *The American journal of pathology* 155.5 (1999), pp. 1467–1471. ISSN: 00029440. DOI: 10.1016/S0002-9440(10)65461-2.

[22] V Zsikla, M Baumann, and G Cathomas. "Effect of buffered formalin on amplification of DNA from paraffin wax embedded small biopsies using real-time PCR." In: *Journal of clinical pathology* 57.6 (2004), pp. 654–656. ISSN: 0021-9746. DOI: 10.1136/jcp.2003.013961.
